# Regulators of AWC-Mediated Olfactory Plasticity in *Caenorhabditis elegans*

**DOI:** 10.1371/journal.pgen.1000761

**Published:** 2009-12-11

**Authors:** Damien M. O'Halloran, Svetlana Altshuler-Keylin, Jin I. Lee, Noelle D. L'Etoile

**Affiliations:** 1 Center for Neuroscience, University of California Davis, Davis, California, United States of America; 2 Department of Psychiatry and Behavioral Sciences University of California Davis, Davis, California, United States of America; University of California San Diego, United States of America

## Abstract

While most sensory neurons will adapt to prolonged stimulation by down-regulating their responsiveness to the signal, it is not clear which events initiate long-lasting sensory adaptation. Likewise, we are just beginning to understand how the physiology of the adapted cell is altered. *Caenorhabditis elegans* is inherently attracted to specific odors that are sensed by the paired AWC olfactory sensory neurons. The attraction diminishes if the animal experiences these odors for a prolonged period of time in the absence of food. The AWC neuron responds acutely to odor-exposure by closing calcium channels. While odortaxis requires a Gα subunit protein, cGMP-gated channels, and guanylyl cyclases, adaptation to prolonged odor exposure requires nuclear entry of the cGMP-dependent protein kinase, EGL-4. We asked which candidate members of the olfactory signal transduction pathway promote nuclear entry of EGL-4 and which molecules might induce long-term adaptation downstream of EGL-4 nuclear entry. We found that initiation of long-term adaptation, as assessed by nuclear entry of EGL-4, is dependent on G-protein mediated signaling but is independent of fluxes in calcium levels. We show that long-term adaptation requires polyunsaturated fatty acids (PUFAs) that may act on the transient receptor potential (TRP) channel type V OSM-9 downstream of EGL-4 nuclear entry. We also present evidence that high diacylglycerol (DAG) levels block long-term adaptation without affecting EGL-4 nuclear entry. Our analysis provides a model for the process of long-term adaptation that occurs within the AWC neuron of *C. elegans*: G-protein signaling initiates long-lasting olfactory adaptation by promoting the nuclear entry of EGL-4, and once EGL-4 has entered the nucleus, processes such as PUFA activation of the TRP channel OSM-9 may dampen the output of the AWC neuron.

## Author Summary

*Caenorhabditis elegans* is capable of sensing a variety of attractive volatile compounds. These odors are the worm's “best guesses” as to how to track down food. Employing calculated approximations underlies a foraging strategy that is open to failure. When *C. elegans* track an odor which proves unrewarding, they must modify their behavior based on this experience. They also need to prevent over-stimulating their neurons. To accomplish this, *C. elegans* olfactory sensory neurons adapt to odors after a sustained exposure to odor in the absence of food. Within the pair of primary odor-sensory neurons, termed the AWCs, adaptation requires the cGMP-dependent protein kinase G (PKG), EGL-4. Exposing animals to AWC–sensed odors for approximately 60 minutes results in a long-lasting (∼3 hour) adaptation that requires the nuclear translocation of EGL-4. To understand how sensory transduction and desensitization machinery converge to achieve olfactory adaptation, we asked whether odor-induced EGL-4 nuclear accumulation was affected by gene mutations that abrogate either odor sensation of or adaptation to AWC–sensed odors. We find that G-protein signaling represents the integration point where primary odor sensation and odor adaptation pathways diverge. PUFA signaling, calcium, and decreased diacylglycerol all dampen the response of the AWC neuron to odor downstream of this divergence.

## Introduction

Olfactory adaptation may subserve a food-seeking strategy in *C. elegans*. Since the sources of many odors that are inherently attractive to *C. elegans* do not, in fact, provide a source of nutrition, the worm may have to discriminate between rewarding and unrewarding stimuli by adapting to nutritionally profitless odors. This modification in olfactory behavior, as a function of experience, represents a finely regulated signaling circuit [1]–[8].

**Figure 1:**
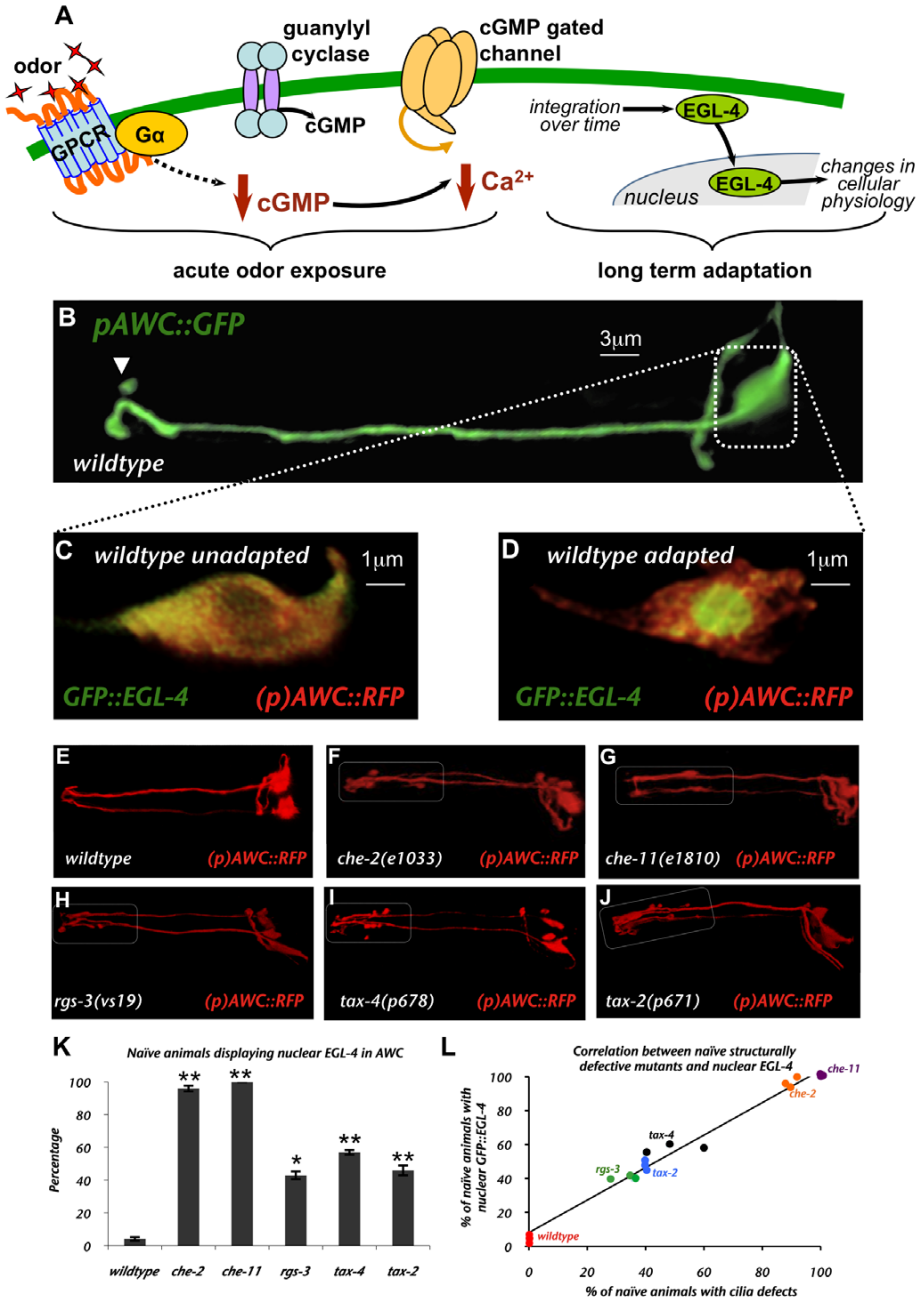
Integrity of AWC cilia structure is critical for proper localization of EGL-4. (A) A model of olfactory signaling and adaptation in AWC. Odor is thought to bind directly to olfactory G-protein coupled receptors [10],[27]. Calcium levels in AWC have been shown to decrease after ligand binding [13]. This is likely regulated by the cGMP-gated calcium channels TAX-2 and TAX-4 [14],[15]. cGMP levels are likely decreased by odor binding and the activation of the Gα protein, ODR-3. This signal is integrated over time resulting in nuclear localization of the protein kinase G, EGL-4. The nuclear entry of EGL-4 causes changes in the cellular physiology of AWC and is both necessary and sufficient for AWC adaptation [26]. (B) Fluorescent confocal image of an AWC neuron highlighting the cell body area captured in images (C) and (D). (C) Deconvolved confocal image of AWC from a naive GFP::EGL-4 expressing worm with GFP::EGL-4 throughout the AWC cytosol. (D) Deconvolved confocal image of AWC from an adapted GFP::EGL-4 expressing animal. GFP::EGL-4 can be seen accumulated inside the AWC nucleus. Yellow color in (C) and (D) indicates co-localization of both GFP and RFP in AWC. Fluorescent confocal images of AWC in (E) wildtype, (F) *che-2(e1033)*, (G) *che-11(e1810)*, (H) rgs-3(vs19), (I) tax-4(p678) and (J) tax-2(p671). Morphological defects indicated by white dotted boxes are observed along the AWC dendritic process and/or at the AWC cilia. (K) Percentage of animals exhibiting GFP tagged EGL-4 constitutively in the AWC nucleus of naive animals. ** Indicates *p*≤0.005 and * indicates *p*≤0.05 significant differences between mutants and wildtype animals. (L) Correlation between cilia defects and nuclear GFP::EGL-4 in naïve *che-2*, *che-11*, *rgs-3*, *tax-4*, and *tax-2* animals. Pearson correlation coefficient for the cilia defects and percent naïve worms with nuclear GFP::EGL-4 was 0.99 with p<0.0001. Error bars represent the S.E.M. For all images anterior is to the left.

The AWC cilia contain olfactory receptors that enable the nematode to sense inherently attractive volatile odors [9],[10]. These olfactory receptors are coupled intracellularly to Gα subunits [11],[12]. Presentation of an odor results in decreased intracellular calcium levels [13] which is likely mediated by closing of the cGMP-gated channels, TAX-2 and TAX-4 [14],[15]. cGMP is produced by the guanylyl cyclases, ODR-1 and DAF-11 [16],[17]. A schematic of the signal transduction cascade is presented in Figure 1A. Following this schema, odor sensation by the AWC neurons is likely to mirror light sensation by vertebrate rod cells in that both utilize a GPCR and cGMP as the second messenger [18],[19]. Both light in the rod cell and odor in the AWC decrease intracellular calcium levels to hyperpolarize the cells when stimulated and both cell types tonically release neurotransmitter to the downstream interneurons [13],[18],[19].

The genetics of olfactory adaptation were first investigated in *C. elegans* by Colbert and Bargmann [1]. At that time, the authors defined parameters of the olfactory adaptation paradigm, which they used to design a genetic screen to isolate mutants defective in their ability to adapt to volatile odors sensed by the AWC sensory neurons. Mutants identified were the as yet uncloned *adp-1(ky20)*, which fails to adapt to the AWC sensed odors benzaldehyde and butanone, and the TRPV channel mutant *osm-9(ky10)*, which is defective in adaptation to the AWC sensed odors butanone and isoamyl alcohol. The Bargmann group and other laboratories have since identified additional mutants that are unable to adapt to AWC-sensed odors; these mutants include: the arrestin *arr-1* [5], the T-box transcription factor *tbx-2* [20], the calcium dependent calcineurin A phosphatase subunit *tax-6*, which actually down regulates adaptation [21], the RNA binding PUF protein FBF-1 [8] which increases local translation of EGL-4, the calsyntenin/alcadein ortholog CASY-1 [22], the Ras-MAPK pathway which functions at the interneuron AIY [23], the GPC-1 γ subunit which regulates a rapid form of adaptation termed ‘”early adaptation” [24], the guanylyl cyclase GCY-28 which regulates odor preference and odor-exposure induced avoidance of butanone [25], and the G_o_α/G_q_α DAG signaling mutants [4]. The cGMP-dependent protein kinase (PKG) EGL-4 has also been shown to be necessary at the time of odor exposure for adaptation to all AWC-sensed odors [3],[8],[26].

Examination of the temporal sequence of events leading up to long-term adaptation revealed that it occurs in at least two phases: an initial “early” adaptation within the first 10 minutes of exposure [23], then a rapidly reversible phase that results after 30 minutes of odor exposure (referred to as short-term adaptation) and subsequently, a longer, more enduring phase that results after 60 minutes of exposure (called long-term adaptation) [3],[26]. In the wildtype animal, EGL-4 acts within the cytoplasm to promote short-term adaptation [3] and in the nucleus to promote long-term adaptation to AWC sensed odors [26]. Further, we showed in Lee et al. [26] that in the wildtype animal under normal laboratory conditions, nuclear accumulation of EGL-4 is both necessary and sufficient to evoke long-term olfactory adaptation. Though changes within the whole olfactory circuit, including the interneurons AIY and AIB, are likely to occur during each phase of the process of adaptation, this work will focus on long-term adaptation and the events that take place within the AWC sensory neuron.

To determine which step along the olfactory signal transduction pathway is integrated over time to evoke long-term adaptation, we examined olfactory behavior as well as nuclear accumulation of a GFP-tagged form of EGL-4 (GFP::EGL-4) (Figure 1D and [26]) in genetic backgrounds where either signal transduction or adaptation is perturbed (Table S1 and Figure S1). Thus, we used the visible accumulation of GFP::EGL-4 within the AWC nucleus as a hallmark of one aspect of long-term adaptation. Using this approach, we found that nuclear accumulation of GFP::EGL-4 is independent of calcium levels but dependent on Gα protein activity. Our visible readout for this important aspect of long-term odor adaptation also allowed us to place novel as well as previously identified olfactory adaptation-defective mutants either downstream of EGL-4 nuclear entry or into a parallel genetic pathway that works with EGL-4 to promote adaptation. For example, we show that the Polyunsaturated Fatty Acids (PUFAs) are novel regulators of olfactory adaptation that may act downstream of EGL-4's nuclear entry along with the TRP channel, OSM-9.

## Results

### Mutants with morphologically defective sensory cilia exhibit constitutively nuclear EGL-4

Signal transduction in the primary sensory neuron AWC begins at the most distal portion of the dendrite, in the sensory cilia (arrowhead in Figure 1B). Since signaling molecules such as GPCRs [10],[27] , guanylyl cyclases [16] and the G-protein α subunits [11],[28] localize to the sensory cilia, it is likely that sensory signaling requires an intact cilia structure. Thus, we asked how the structural integrity of the AWC cilia might affect EGL-4's localization.

In the naïve animal, GFP::EGL-4 is evenly distributed throughout the cytosol of the AWC and after prolonged odor exposure it redistributes to the nucleus (Figure 1C and 1D and [26]). We examined the distribution of GFP::EGL-4 in mutants with highly transmitted ciliopathies: *che-2(e1033)* and *che-11(e1810)* [29],[30]. CHE-2 is a WD-40 intraflagellar transport (IFT) complex B protein [30] and CHE-11 is a homolog of the *Chlamydomonas* IFT complex A protein [29],[31]. Both IFT complex A and B genes are necessary for ciliogenesis in *C. elegans* [31]. The *e1033* allele of *che-2* is a predicted null [30]. Cilia defects were identified by examining the integrity of AWC structure using the transcriptional reporter, (*p)odr-1*::RFP, which expresses soluble RFP throughout AWC (Figure 1E–1J). The *che-2(e1033)* and *che-11(e1810*) mutants displayed AWC cilia defects in nearly all animals examined (Figure 1F and 1G). We found that EGL-4 was in the AWC nuclei of close to 100% of either naïve *che-2(e1033*) or naïve *che-11(e1810*) mutant animals (Figure 1K). Each and every cilia-defective animal of either genotype displayed nuclear EGL-4 while cytoplasmic EGL-4 was only observed in animals with wildtype cilia.

We also observed nuclear EGL-4 in naive animals with mutations in genes encoding: *rgs-3(vs19) –* a regulator of G protein signaling protein [32]; *tax-4(p678) – a* cyclic nucleotide gated channel alpha subunit [15] and *tax-2(p671) – a* cyclic nucleotide gated channel beta subunit [14]. When we examined the morphology of the AWCs in these mutant animals we found that they displayed severe defects at the distal portion of the dendrite and cilia as indicated by white dotted boxes (Figure 1H–1J). These defects were similar to those observed in the *che-2(e1033)* and *che-11(e1810)* mutants (Figure 1F–1G). The penetrance of these defects is shown in Figure 1K. We asked whether there was a correlation between cilia defects and the incidence of nuclear EGL-4 in naïve *che-2(e1033)*, *che-11(e1810)*, *rgs-3(vs19)*, *tax-2(p671)* or *tax-4(p678)* mutant worms. We found a strong correlation (Pearson's correlation coefficient, *r* = 0.99, with a p value of <0.0001) between the penetrance of the cilia defects and the incidence of nuclear GFP::EGL-4, regardless of the genotype (Figure 1L).

**Figure 2:**
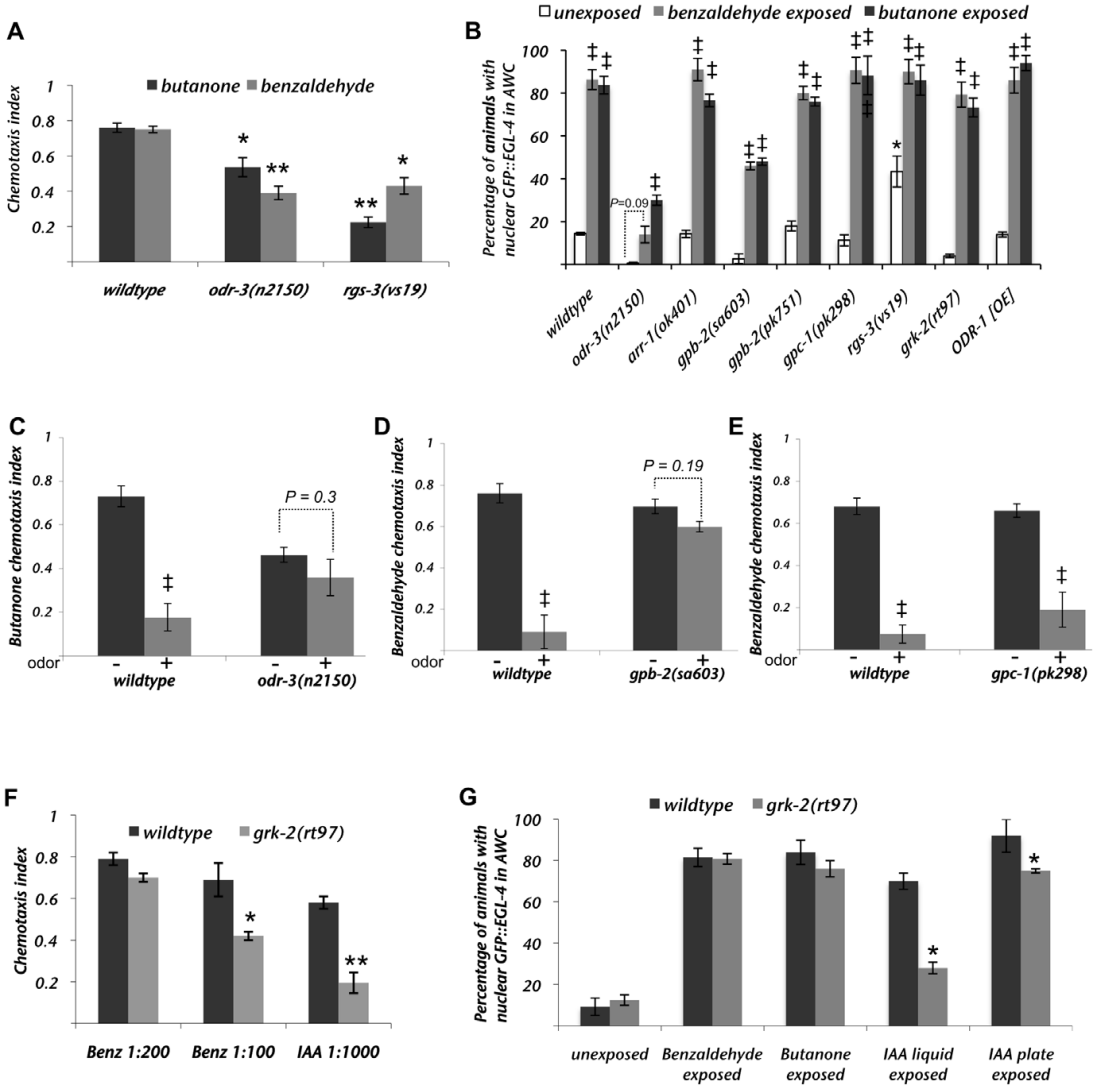
G-alpha signaling is required both for odor-induced nuclear accumulation of EGL-4 and for adaptation to the AWC–sensed odor butanone. (A) Chemotaxis responses of *odr-3* and *rgs-3* mutants to the AWC sensed odors, benzaldehyde and butanone. (B) Percent of animals displaying nuclear GFP::EGL-4 in AWCs of naive (white bars), benzaldehyde exposed (black bars) or butanone exposed (gray bars) populations. (C–E) The chemotaxis response of *odr-3(n2150)*, *gpb-2(sa603)* or *gpc-1(pk298)* strains to the odor indicated on the y axis. Odor pre-exposure (“+”) is unable to adapt the chemotaxis response of either the *odr-3(n2150)* or *gpb-2(sa603)* strains: compare odor pre-exposed “+” with buffer exposed “−” populations. The *gpc-1(pk298)* strain, however, adapts like wild-type. (F) Chemotaxis responses of wildtype (black bars) or *grk-2(rt97)* mutant (gray bars) animals. (G) Percent of wildtype or *grk-2(rt97)* animals in a population that displayed nuclear GFP::EGL-4 in AWC after exposure to either buffer alone (unexposed) or the indicated odor. Error bars represent the S.E.M. ** Indicates a *p*≤0.005 and * indicates a *p*≤0.05 for the differences between mutants and wildtype animals. ^‡^ Indicates a *p*≤0.05for the difference between unexposed and odor-exposed populations. *p* values were calculated using the Student's *t*-test.

There are certain exceptions to these findings. The Gα signaling *odr-3* mutant strain was shown to have defective AWC cilia (*odr-3(n1605)* allele - [11]) but when we examined the *n2150* allele we found it exhibited wildtype subcellular localization of GFP::EGL-4 in naive animals (Figure 2B). This may be because the cilia and distal region of the dendrite in the *n2150* allele of *odr-3* are less severely affected than in *odr-3(n1605)* and the *che-2(e1033)*, *che-11(e1810)*, *rgs-3(vs19)*, *tax-2(p678)* and *tax-4(p671)* strains we examined (Figure S4). We observed severe morphological defects in both the cilia and the distal region of the dendrite of the latter mutants. Specifically, we observed misdirected extensions, which failed to extend as far anteriorly as wildtype cilia. The area affected is indicated by the white dotted boxes in Figure 1F–1J. The distal region of the dendrites of *odr-3(n2150)*, however, were intact and the cilia resembled the more condensed fork-shaped cilia of AWB (Figure S4). Indeed, electron microscopic analysis of this allele's cilia corroborate these findings [9]. Conversely, though the guanylyl cyclase signaling defective *odr-1(n1936)* mutant strain has visually intact AWC cilia [16], it exhibited 100% nuclear GFP::EGL-4 in naive worms (data not shown). One explanation is that the AWC cilia of *odr-1* mutant worms are non-functional, indeed, Mukhopadhyay et al. [33], showed that the AWB cilia of *odr-1(n1936)* mutant worms were not like those of their well-fed wildtype counterparts; they more closely resembled the cilia of starved animals. This raises the possibility that the AWC cilia are also defective in *odr-1* mutants, perhaps in a way that specifically affects EGL-4 cytoplsamic localization. On the whole, the data suggest that intact cilia/dendrite morphology and perhaps function is required to keep EGL-4 in the AWC cytoplasm of the naïve animal.

### The G alpha subunit ODR-3 is necessary for odor-induced nuclear translocation of GFP::EGL-4

To better understand how odor-exposure adapts AWC-mediated chemotaxis, we asked first how the initial events of sensory signaling might affect odor-adaptation and GFP::EGL-4 nuclear entry. Since AWC-mediated chemotaxis represents the behavioral output from a G-protein coupled receptor (GPCR) initiated signaling pathway (Figure 1A and [10],[11],[27],[28]) we first examined mutants that lack G-protein signaling components and their regulators.

Stimulation of a GPCR causes the Gα subunit to dissociate from the β and γ subunits to initiate second messenger cascades often by activating an adenylyl cyclase to increase or a phosphodiesterase to decrease second messenger levels [34]. The β and γ subunits, however, often play additional regulatory roles [34], such as directing the phosphorylation of the GPCR by a G-protein receptor kinase (*grk*). This phosphorylation leads to recruitment of cytosolic arrestin, which then leads to desensitization of the GPCR by disallowing further Gα binding [5],[35]. Likewise, the activity of the Gα can be inhibited by a regulator of G-protein signaling (RGS) molecule [32]. Thus, the Gα subunit mutant *odr-3(n2150)* [11]; the Gβ subunit mutants *gpb-2(sa603)* and *gpb-2(pk751)* [24]; the Gγ subunit mutant *gpc-1(pk298)* [24]; the RGS mutant *rgs-3(vs19)* [32]; the arrestin mutant *arr-1(ok401)* [5]; and the G-protein receptor kinase mutant *grk-2(rt97)* [35] strains were examined for their ability to induce nuclear localization of GFP::EGL-4 in response to odor exposure. The AWC-mediated chemotaxis responses of *odr-3(n2150)* and *rgs-3(vs19)* are shown in Figure 2A as positive controls that corroborate previous observations with these mutants [11],[32].

We first examined the *odr-3(n2150)* mutant strain. 80 minute exposure to benzaldehyde failed to induce nuclear accumulation of EGL-4 (Figure 2B) as the percent of worms that showed nuclear EGL-4 after benzaldehyde exposure was statistically indistinguishable from either the naïve *odr-3* mutant (*P* = 0.09) or naïve wildtype worms (Figure 2B). This indicates that ODR-3 is necessary for benzaldehyde to induce nuclear accumulation of EGL-4. The small percent of *odr-3* mutant animals observed with nuclear GFP::EGL-4 following butanone exposure (Figure 2B) may reflect the involvement of another Gá in butanone signaling, as was suggested previously [11],[16]. Likewise, chemotaxis to butanone is only slightly compromised (Figure 2A and 2C and [11]). In summary, we find that ODR-3 Gá signaling is necessary for nuclear accumulation of EGL-4. The *odr-3(n2150)* mutant strain is only mildly defective in chemotaxis to butanone and so could be examined for its ability to adapt to this odor. We found that this *odr-3* mutant strain was unable to adapt to butanone (Figure 2C). Thus, ODR-3 is required both for accumulation GFP::EGL-4 in the AWC nucleus and adaptation of the butanone seeking response.

We next examined the contribution of β and γ subunits of the G-protein trimer to odor-induced GFP::EGL-4 nuclear accumulation. There are two known genes encoding the Gβ subunits (*gpb-1* and *gpb-2*) and two known genes encoding the Gγ subunits (*gpc-1* and *gpc-2*) in the *C. elegans* genome. Both *gpc-2* and *gpb-1* mutants are lethal and could not be assayed. The GPB-2 β subunit was previously shown to be required for adaptation to the odor benzaldehyde and we confirmed this result using another predicted null allele, *gpb-2(sa603)* (Figure 2D and [4]). When we examined odor-induced nuclear accumulation of GFP::EGL-4 in this mutant strain, we found that benzaldehyde exposure increased the percentage of *gpb-2(sa603)* animals with nuclear GFP::EGL-4 over that of the naïve population (naïve = 2.7%, exposed = 47%, *p* = 0.030). Though this is significantly different from the percent of wildtype animals that exhibited nuclear GFP::EGL-4 (wildtype = 83.7%, *p* = 0.025), the fact that we saw a significant increase with odor exposure encouraged us to examine the deletion allele of *gpb-2*, *pk751* [36]. We found that *gpb-2(pk751)* did not affect odor-induced nuclear accumulation of GFP::EGL-4 (Figure 2B – fifth set of bars). Thus, the Gβ could be required for adaptation either downstream of EGL-4 nuclear entry or in a pathway parallel to it. It is unlikely, however, to act in the same way as ODR-3. The *gpc-1* γ subunit mutant was neither defective for this long-term adaptation nor defective for odor-induced nuclear accumulation of GFP::EGL-4 (Figure 2E and 2B). This is in contrast to the recent discovery that GPC-1 is required for a very rapid (less than 10 minute exposure) adaptation to benzaldehyde [24]. This suggests that short, (10 minute) and long (80 minute) exposures adapt the AWC olfactory response via different mechanisms.

The regulator of G-protein signaling, RGS-3 [32]; the sole *C. elegans* arrestin, ARR-1 [5] and the G-protein receptor kinase, GRK-2 [35] were also assayed for involvement in EGL-4's odor-induced nuclear accumulation. As was seen previously for the AWC-sensed odor isoamyl alcohol [32], *rgs-3(vs19)* mutants also exhibited reduced chemotaxis to either benzaldehyde or butanone (Figure 2A) at the concentrations of the odor that elicit a peak response in wildtype animals [9]. A significantly higher than wildtype percent of naïve *rgs-3(vs19)* mutant animals exhibited nuclear GFP::EGL-4 (43%, Figure 2B, white bar). This increase could, however, be accounted for by the fact that the animals with nuclear GFP::EGL-4 also had defective cilia (see Figure 1L for the correlation between defective cilia and nuclear GFP::EGL-4 in *rgs-3*). Prolonged odor-exposure was able to further increase the percentage of *rgs-3* animals that exhibited nuclear GFP::EGL-4 (from 43% to 90%, grey and black bars, Figure 2B). Thus, *rgs-3* mutant animals are either more sensitive than, or as sensitive as wildtype animals to prolonged odor exposure as assessed by the ability of odor to induce nuclear accumulation of EGL-4.

ARR-1 has previously been shown to be required for adaptation to AWC-sensed odors [5]. Though the *arr-1(ok401)* mutant strain was unable to adapt to either butanone or benzaldehyde in our hands as well (data not shown), GFP::EGL-4 accumulated within the nuclei of the same percentage of an *arr-1* mutant population of animals as in a wildtype population (Figure 2B). Thus, ARR-1, like GPB-2, may function in parallel to, or downstream of, EGL-4's translocation to the nucleus.

As previously reported [35], mutations in the G-protein receptor kinase *grk-2* gene caused defects in the animals' ability to sense either benzaldehyde (1∶100 dilution) or isoamyl alcohol (1∶1000 dilution) (Figure 2F). These mutant animals, however, were no different in their ability to accumulate GFP::EGL-4 within their nuclei in response to prolonged butanone and benzaldehyde exposure than wildtype animals (Figure 2G). We did, however, observe a defect in *grk-2* mutants with respect to isoamyl alcohol's ability to induce GFP::EGL-4 nuclear accumulation. Whether we soaked the animals in buffer containing dilute isoamyl alcohol or exposed them to the volatile odor while on an unseeded plate, the percentage of *grk-2* animals that showed GFP::EGL-4 accumulation in the AWC nucleus was significantly different from that of the wildtype strain (Figure 2G). That is, after exposure to isoamyl alcohol dispersed in liquid, 28% of *grk-2* and 72.5% of wildtype animals exhibited nuclear GFP::EGL-4 (*p* = 0.011). Likewise, after prolonged exposure to isoamyl alcohol dispersed in air, 75% of *grk-2* and 92% of wildtype animals exhibited nuclear GFP::EGL-4 (*p* = 0.012). Thus, *grk-2* animals would seem to have a lower sensitivity than wildtype animals to prolonged (Figure 2G), as well as acute (Figure 2F), exposure to isoamyl alcohol. This is consistent with findings presented previously that showed that *grk-2* mutants have reduced acute responses in the ASH nociceptive sensory neurons of *C. elegans* to high osmolarity and quinine but not to nose touch [35]. Thus, in AWC as well as in ASH the primary response to certain stimuli may be reduced in *grk-2* mutant animals. Interestingly, nuclear accumulation of EGL-4, which depends on integration of primary sensory signaling over time is also blunted in *grk-2* mutants.

### cGMP and nuclear accumulation of EGL-4

The next step in the signal transduction pathway is regulation of the second messenger, which, in AWC, is thought to be cGMP. Since the calcium current is likely to be mediated by opening of the cGMP-gated TAX-2/4 channel [14],[15],[37] and calcium decreases upon odor stimulation [13], it is also likely that cGMP levels are high in the odor-naive animals and decrease acutely in response to odor-stimulation. In Lee et al. [26], we showed by mutating the cGMP-binding sites within EGL-4, that cGMP binding by EGL-4 is likely required for its ability to accumulate within the AWC nucleus in response to odor. Thus, some level of cGMP is also likely to be required for EGL-4 nuclear accumulation. We asked whether fluxes in cGMP levels were important for odor to induce nuclear accumulation of EGL-4. To attempt to raise the cGMP levels within AWC and thereby to interfere with cGMP fluxes, we exposed worms to the non-cleavable, membrane permeable cGMP analog, 8-Bromo-cGMP (8-Br-cGMP). We soaked animals in or grew animals on plates impregnated with 8-Br-cGMP and asked how this affected the localization of GFP::EGL-4 in either naive or odor-exposed animals. This manipulation had no effect on the localization of EGL-4: it was cytoplasmic in either the 8-Br-cGMP-treated or the untreated naive animals and nuclear in each cohort of odor-exposed animals (Figure S1). Thus, either fluxes in cGMP levels do not affect nuclear accumulation of EGL-4 after prolonged odor-exposure or we were not able to interfere with these fluxes using 8-Br-cGMP. It is difficult to know whether these manipulations are achieving our goal since there is currently no way to directly assess cGMP levels within the AWC neurons of the living animal.

Another approach to interfere with the flux of cGMP is to overexpress a cGMP-producing guanylyl cyclase. At least two guanylyl cyclases, DAF-11 and ODR-1, are known to be expressed in AWC and loss of either cyclase renders the animal unable to seek AWC-sensed odors [16],[17]. Overexpression of ODR-1 is likely to boost cGMP levels in that it leads to a defect in discrimination between butanone and benzaldehyde that is dependent upon an intact cyclase domain [16]. This discrimination defect is, at least in part, due to the fact that both AWC neurons respond to butanone [38]. It also causes other potentially cyclase-independent defects that render animals unable to adapt to butanone [16]. In our present studies, we found that over-expression of ODR-1 did not affect GFP::EGL-4 localization: GFP::EGL-4 was cytoplasmic in the naive animals and nuclear in the butanone-exposed ones (Figure 2B and Figure S1 – ODR-1[OE] bars). Importantly, we could confirm that ODR-1 was overexpressed as the animals were unable to adapt to butanone (Figure S2) and they showed nuclear GFP::EGL-4 in both AWCs of roughly 50% of the butanone-exposed animals (data not shown). This is consistent with the fact that approximately 50% of ODR-1 overexpressing animals have two STR-2 ‘ON’ (and therefore two butanone-responsive) AWC neurons [38] and that butanone only sends GFP::EGL-4 into the STR-2 ‘ON’ AWC neuron [26]. Further, these animals were insensitive to 2,3 pentanedione (Figure S2A) as would be predicted if both AWCs were STR-2 ‘ON’ (butanone-responsive) and neither was STR-2 ‘OFF’ (2,3 pentanedione responsive) [38]. Thus, potentially interfering with the cGMP fluxes by overexpressing ODR-1 does not interfere with odor's ability to send EGL-4 into the AWC nucleus. This would indicate either that we are not able to adequately block fluxes in cGMP using these methods or that cGMP-binding is not sufficient to induce nuclear localization of EGL-4 in response to odor. EGL-4 might act as a coincident detector to integrate the presence of cGMP with a second perhaps Gα derived signal. The potentially cGMP-independent adaptation defects incurred by overexpressing ODR-1 must occur either downstream of or in parallel to EGL-4 nuclear entry since the animals that overexpress ODR-1 fail to adapt to butanone even though EGL-4 is accumulated within their nuclei.

### The dynamic odor-induced nuclear translocation of EGL-4 is independent of calcium signaling

**Figure 3:**
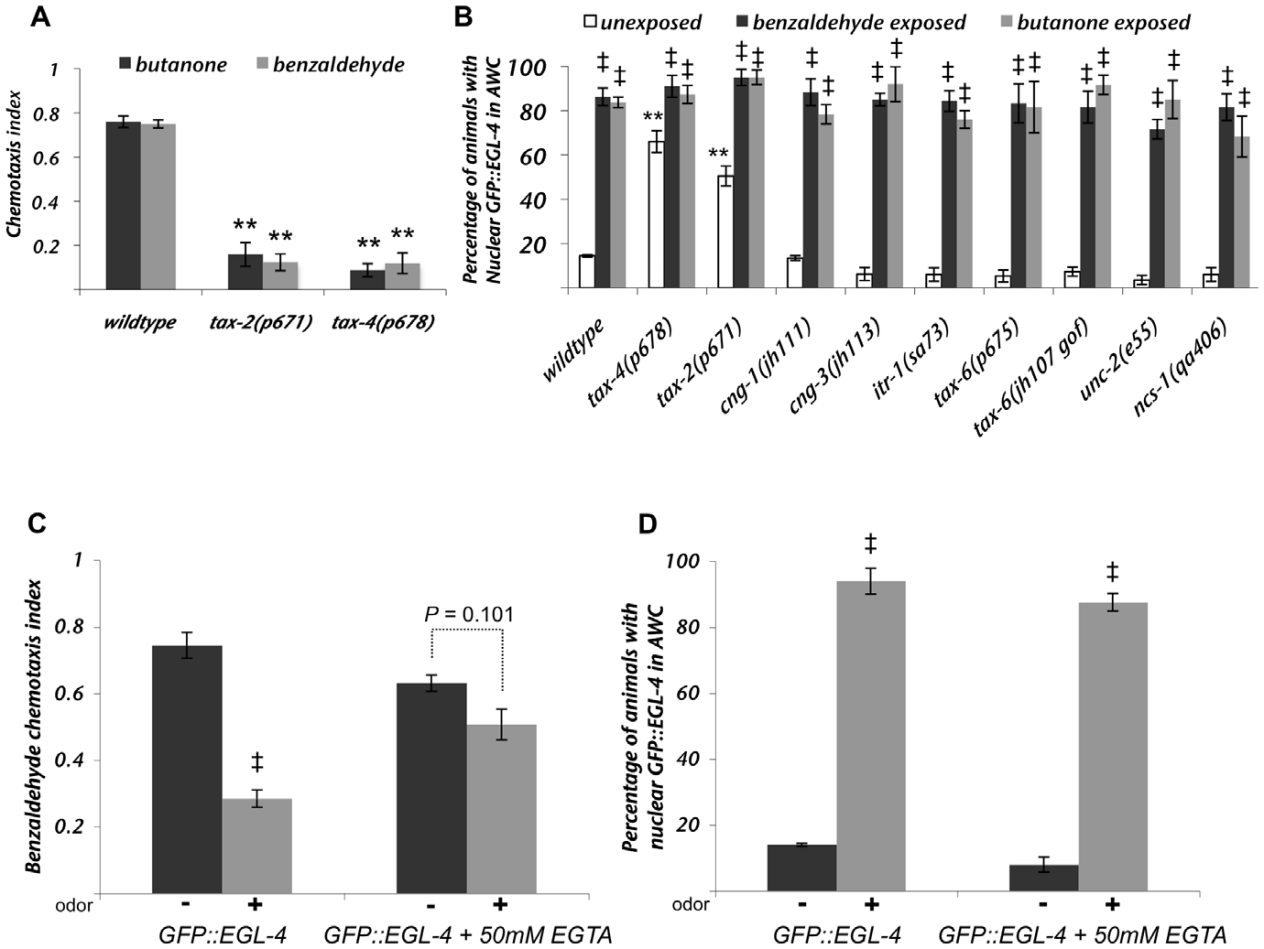
Calcium signaling does not regulate the odor-induced nuclear accumulation of EGL-4. (A) Chemotaxis responses of *tax-2* and *tax-4* mutant strains to the AWC–sensed odors benzaldehyde (gray bars) and butanone (black bars). (B) Calcium signaling mutants expressing GFP-tagged EGL-4 from an integrated array (*pyIs500*) were exposed to buffer containing either no odor (un-exposed, white bars) or benzaldehyde (black bars) or butanone (gray bars) for 80 minutes before microscopic examination of localization of GFP::EGL-4 within the AWC neurons of each population of animals. (C and D, bars on left side of each graph) GFP tagged EGL-4 expressing animals were exposed to either buffer alone (“−” black bars) or buffer plus benzaldehyde (“+” gray bars) for 80 minutes. (C and D, bars on the right side of graphs) represent populations of animals that were treated in a similar way as those on the left except that they had been pre-exposed for 2 hours to 50mM of the calcium chelator, EGTA. Exposure to EGTA inhibited adaptation of the odor-seeking response (C) but not the nuclear accumulation of GFP::EGL-4 (D). Error bars represent the S.E.M. ** Indicates *p*≤0.005 and * indicates *p*≤0.05 significant differences between mutants and wildtype animals. ^‡^ Indicates significant difference at *p*≤0.05 between unadapted and adapted mutant bars or unadapted and adapted wildtype bars. *p* values were calculated using the Student's *t*-test.

Calcium levels in AWC are critical for acute odor responses ([1],[14],[15] and Figure 3A). G-protein signaling in AWC mediates an odor-induced decrease in calcium [13]. Thus, decreases in calcium might be predicted to induce adaptation. Calcium, however, has been shown to be required for olfactory adaptation by Colbert and Bargmann [1] and is known to be critical for synaptic vesicle release. Further, in other systems, calcium increases are a key integrator of signaling events [39]–[41]. To understand whether calcium is required for nuclear translocation of EGL-4, we examined GFP::EGL-4 localization in the mutant strains that affect different aspects of calcium signaling (Figure 3B). The calcium signaling proteins surveyed include the cGMP-gated calcium channel subunits, TAX-4, TAX-2, CNG-1 and CNG-3 [14],[15],[42],[43], the internal calcium store regulator ITR-1 [44], the calcineurin A protein subunit TAX-6 [21], the voltage gated calcium channel UNC-2 [45],[46] and NCS-1, an AWC-expressed calcium sensor [47]. None of these calcium-signaling proteins were required for the nuclear accumulation of GFP::EGL-4 after prolonged odor exposure (Figure 3B). The cyclic nucleotide gated channel subunits TAX-2 and TAX-4 are required for AWC-mediated chemosensation (Figure 3A, [14],[15]) and their loss caused GFP::EGL-4 to localize within the AWC nuclei of naive animals (Figure 3B – second and third set of white bars, 70% and 50%). However, nuclear GFP::EGL-4 in naïve *tax-2/4* mutant animals was correlated with ciliopathies in the same way that was observed for *rgs-3* (Figure 1L). As with the *rgs-3* mutants, the percentage of *tax-2* or *tax-4* animals that displayed nuclear GFP::EGL-4 increased significantly after prolonged odor exposure (Figure 3B – white bars versus black and grey bars for *tax-2* and *tax-4*, *p*<0.05 between unexposed and pre-exposed populations). This suggests that odor was still able to induce nuclear accumulation of GFP::EGL-4 even in the absence of TAX-2/TAX-4 mediated calcium fluctuations. To ask whether the nuclear accumulation of EGL-4 might be a direct consequence of decreased calcium, we incubated GFP::EGL-4 expressing animals with odor and the calcium chelator, ethylene glycol tetraacetic acid (EGTA). We found that though EGTA was effective enough to blunt adaptation of the chemotaxis response in wildtype animals (Figure 3C - *p* = 0.101 for buffer versus buffer plus odor pre-exposure after EGTA treatment), it was unable to induce nuclear accumulation of EGL-4 in odor-naïve animals or perturb the nuclear accumulation of EGL-4 in odor-exposed animals (Figure 3D - both EGTA- and non EGTA-treated populations showed significantly different percentages of animals with nuclear GFP::EGL-4 following prolonged odor as compared with buffer exposure, *p*<0.005). Taken together, our analysis of mutants defective in calcium signaling (Figure 3B) along with the lack of effect of EGTA on EGL-4 localization (Figure 3C and 3D), suggests that reduction in calcium levels is not the signal that is integrated over time to trigger EGL-4's nuclear accumulation.

**Figure 4:**
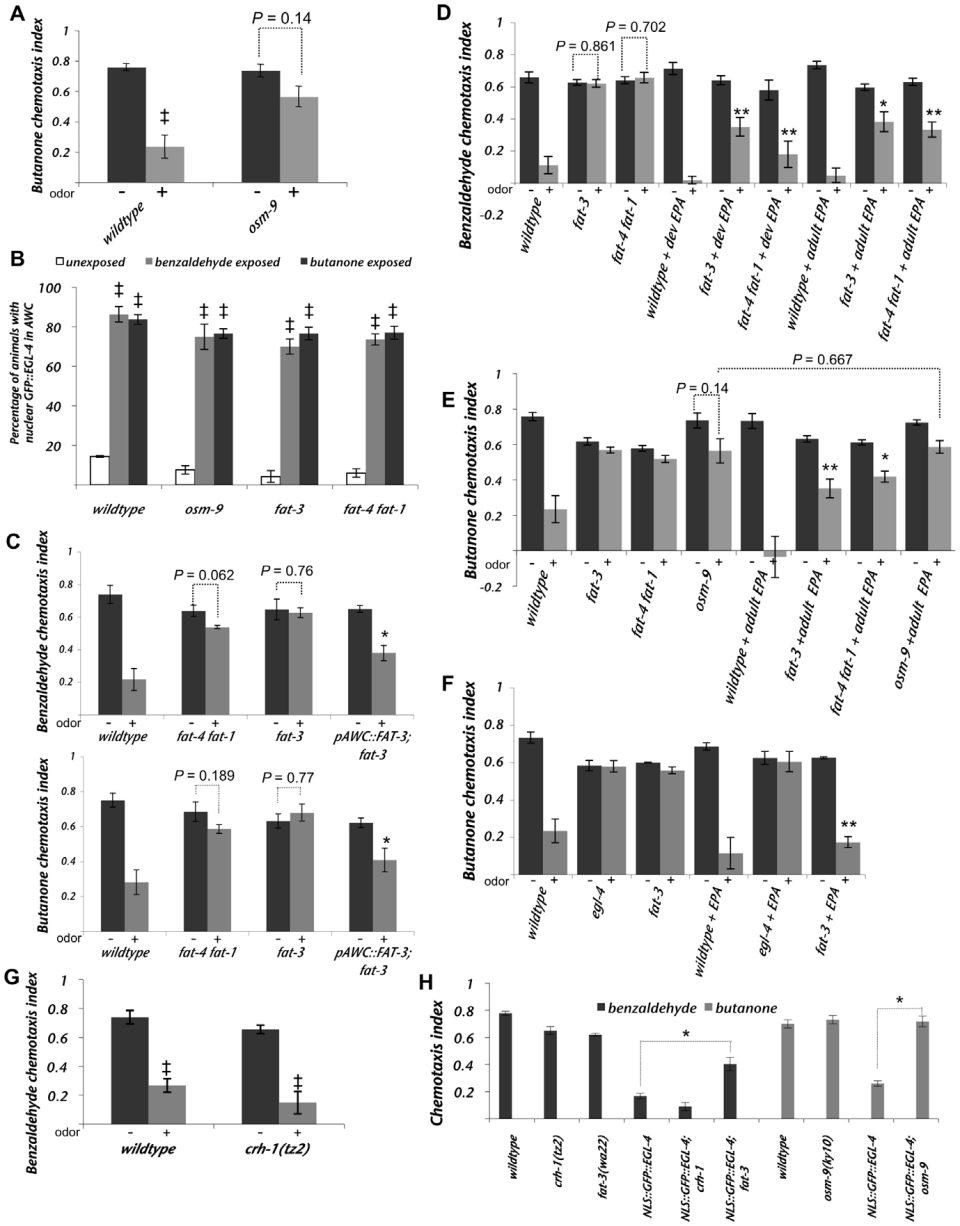
Polyunsaturated fatty acid biosynthesis is required for olfactory adaptation of the AWC neurons. (A) The TRPV channel OSM-9 is required for adaptation to butanone, as previously described [1]. ^‡^ Indicates a difference with *p*≤0.05 between buffer exposed and buffer plus odor exposed populations. (B) The lipid signaling mutants *osm-9*, *fat-3* and *fat-4 fat-1* do not perturb the nuclear accumulation of GFP::EGL-4 after prolonged odor exposure. ^‡^ Indicates a difference with *p*≤0.05 between buffer exposed and buffer plus odor exposed populations. (C) The PUFA synthesis mutants *fat-4(wa14) fat-1(wa9)* and *fat-3(wa22*) displayed defects in their ability to adapt their benzaldehyde (top graph) or butanone (bottom graph) odor-seeking responses. The adaptation defects of *fat-3(wa22)* were significantly rescued by expressing FAT-3 encoding cDNA from an AWC-exclusive promoter (called *(p)AWC::FAT-3::GFP*. This is a shortened form of the *ceh-36* promoter, please see Materials and Methods for details). * indicates a statistically significant (*p*<0.05) difference between the odor-prexposed (“+”) *fat-3* mutant population and *fat-3;Ex[(p)AWC::FAT-3::GFP]* odor-prexposed (“+”) population. (D) Dietary supplementation of the PUFA, EPA, throughout development (dev EPA bars) or 24 hours prior to assaying for adaptation (adult EPA bars) rescued the behavioral adaptation defects of *fat-3(wa22)* and *fat-4(wa14) fat-1(wa9)* animals. * (*p*<0.05) and ** (*p*<0.005) indicate differences between the chemotaxis indices of odor-prexposed (“+”) populations with and without EPA treatment. (E) Dietary supplementation of EPA 24 hours prior to assaying for adaptation (adult EPA bars) rescues the butanone adaptation defect of *fat-3(wa22)* and *fat-4(wa14) fat-1(wa9)* animals but fails to rescue the butanone adaptation defect of *osm-9(ky10)* null animals. * (*p*<0.05) and ** (*p*<0.005) indicate differences between the chemotaxis indices of odor-prexposed (“+”) populations with and without EPA treatment. (F) Dietary supplementation of the PUFA EPA throughout development does not rescue the butanone adaptation defects of *egl-4* null mutant animals. ** indicates a difference (*p*<0.005) between the chemotaxis indices of odor-prexposed (“+”) populations with and without EPA treatment. (G) The CREB mutant *crh-1* strain adapted like the wild-type to benzaldehyde and thus served as a negative control for Figure 4H. ^‡^ indicates difference (*p*≤0.05) between buffer and buffer plus benzaldehyde exposed populations. (H) A constitutively nuclear gain-of-function allele of *egl-4* (NLS::GFP:: EGL-4. The transgene is called Ex[(p)*odr-3*::NLS::GFP::EGL-4]) was generated by appending an extra nuclear localization sequence to the N terminus of EGL-4. Animals expressing this form of EGL-4 responded to odor like odor pre-exposed animals and thus may be constitutively adapted [26]. The CREB mutant *crh-1* was used as a negative control (see 4G). The chemotaxis behavior to benzaldehyde (black) or butanone (gray) of animals expressing the constitutively nuclear NLS::GFP::EGL-4 in either a wild-type, a *fat-3(wa22)* or an *osm-9(ky10)* genetic background is shown. Loss of either FAT-3 (6^th^ bar) or OSM-9 (10^th^ bar) suppressed the constitutively adapted phenotype of NLS::GFP::EGL-4 animals. * indicates the difference (*p*<0.05) between wild-type (N2); Ex[(p)*odr-3*::NLS::GFP::EGL-4] and *fat-3(wa22)*; Ex[(p)*odr-3*::NLS::GFP::EGL-4], *osm-9(ky10)*; Ex[(p)*odr-3*::NLS::GFP::EGL-4] or *crh-1(tz2)*; Ex[(p)*odr-3*::NLS::GFP::EGL-4] chemotaxis indices. For all experiments animals were exposed to odor for 80 minutes and then tested for chemotaxis to a point source of the same odor. “+” bars indicate odor exposed and “−” bars denote buffer exposed animals. Error bars represent the S.E.M. ** Indicates *p*≤0.005 and * indicates *p*≤0.05 significant differences. *p* values were calculated using the Students *t* test.

Further, though nuclear EGL-4 was sufficient to cause adaptation in untreated animals ([26] and Figure 4H, NLS::GFP::EGL-4 bars), the EGTA treatment blocked adaptation of the odor-seeking response (Figure 3C, +EGTA bars) in animals that had nuclear EGL-4 (Figure 3D, +EGTA bars). This indicates that nuclear EGL-4, though sufficient to induce adaptation of the behavioral response at physiological calcium concentrations, is unable to promote adaptation in the absence of calcium. One interpretation of this is that calcium is required either down-stream of or in parallel to EGL-4 nuclear entry to induce adaptation of the odor-seeking behavioral response in a wildtype animal.

### EGL-4's nuclear entry is independent of the TRPV channel OSM-9

Though the TRPV channel OSM-9 was shown previously to be required for adaptation to butanone (Figure 4A and [1],[48]), its role in this process has remained obscure. Hints about its function came from the observation that loss of OSM-9 was able to suppress the hyperadaptation phenotype of the *tax-6* calcineurin mutant [21], which might place it downstream of calcium mediated down-modulation of adaptation. Thus, we wanted to place *osm-9* in a pathway relative to odor-induced EGL-4 nuclear accumulation. When we expressed our GFP::EGL-4 reporter in the *osm-9(ky10)* mutant background, we found that though these animals failed to adapt to the odor butanone (Figure 4A), they displayed normal nuclear localization of EGL-4 after prolonged odor exposure (Figure 4B – second set of bars). Thus, the adaptation defect of the *osm-9* strain is not a consequence of the failure to accumulate EGL-4 in the AWC nucleus. Rather, it could be a consequence of the loss of a separate parallel pathway or loss of a process downstream of EGL-4's nuclear accumulation. It is nearly impossible to perform the double mutant analysis needed to ask whether *egl-4* and *osm-9* are likely to act in the same pathway since *egl-4(n479, null)* mutants are so severely defective for adaptation (Figure 4F and [3]) that one would not be able to observe enhancement in an *osm-9(ky10);egl-4(n479)* strain. Therefore, we examined the role of the possible regulators of TRP channel function, the Polyunsaturated Fatty Acids (PUFAs).

### Polyunsaturated fatty acid synthesis within the AWC neuron is required for olfactory adaptation

Since PUFAs were shown to function through OSM-9 in nociceptive neurons [49], we decided to determine if PUFA biosynthesis is required for adaptation in AWC. The desaturases FAT-1, FAT-3 and FAT-4 along with the elongases ELO-1 and ELO-2 are responsible for the production of twenty carbon PUFAs [49]–[51]. Loss of both FAT-1 and FAT-4 leads to the build up of the 20 carbon dihomo-gamma linolenic acid (DGLA) while the *fat-3* mutant accumulates the 18 carbon linoleic acid [50],[51]. We found that the *fat-4(wa14) fat-1(wa9)* double mutant animals and *fat-3(wa22)* single mutant animals displayed defects in adaptation of AWC-mediated responses (Figure 4C).

To investigate whether PUFA signaling is required in the AWC neuron itself, we expressed FAT-3 tagged with GFP under the control of an AWC-specific promoter (*(p)ceh-36*::FAT-3::GFP see Materials and Methods for promoter details) in a *fat-3*(*wa22*) mutant background. This transgenic line was able to adapt to benzaldehyde odor exposure significantly better than the parental *fat-3(wa22)* mutant strain (Figure 4C – fourth set of bars; *p*<0.05 between buffer and buffer plus odor exposed populations, also *p*<0.05 for *fat-3* odor exposed population versus *fat-3;Ex[pAWC::FAT-3]* exposed population). This indicates that FAT-3 is likely to act within the AWC neurons to promote adaptation.

To determine whether PUFAs are required developmentally or during the process of adaptation, we used dietary supplementation of the desaturase defective strains to provide PUFAs at defined periods of the animal's life. Unlike mammals, *C. elegans* is capable of synthesizing all of its required PUFAs by desaturating and elongating the saturated fats it receives from its microbial diet. This obviates the necessity of ingesting any essential fatty acids [50],[51]. However, work from Khan-Kirby *et al.* [49] showed that dietary supplementation of *fat-3(wa22)* and *fat-4(wa14) fat-1(wa9)* mutant animals with the 20-carbon PUFA eicosapentaenoic acid (EPA) was able to restore chemosensory behavior. To ask whether the adaptation defects of the desaturase defective mutants could be restored by such dietary supplementation either during their development or in adulthood immediately prior to odor-exposure, we prepared Nematode growth media (NGM) plates containing 160ìM EPA, seeded with OP50 *E. coli*, then grew *C. elegans* on these plates prior to collection for assays. By supplementing their diet with EPA throughout development, we were able to rescue the adaptation behavioral defects of *fat-3* and *fat-4 fat-1* animals (Figure 4D – dev EPA bars). As a control, wildtype animals were similarly grown on EPA NGM plates and these animals displayed normal odortaxis behavior, demonstrating that EPA is not sufficient to induce adaptation but is necessary for proper adaptation of the AWC-mediated chemosensory response (Figure 4D).

To determine whether PUFAs are utilized dynamically or developmentally to allow for normal olfactory adaptation, we grew PUFA synthesis-defective mutants on plates without EPA and transferred the animals as young adults to plates with EPA 24 hours prior to assaying for adaptation. In this experiment, *fat-3* mutants were able to adapt significantly better than the untreated *fat-3* controls to odor pre-exposure (C.I. of benzaldeyde exposed, untreated *fat-3* was 0.62, while the benzaldeyde exposed adult EPA-treated *fat-3* mutant strain exhibited a C.I. of 0.38, *p* = 0.011). Though EPA treatment did not rescue the mutant strain's adapted C.I. back to that of wildtype (C.I. wildtype untreated = 0.11 and C.I. *fat-3* dev EPA = 0.35, *p* = 0.02), the EPA treatment was as effective in the adult as if the animals had been exposed to EPA for their entire lives (*p* = 0.723 comparing adult to developmental EPA for *fat-3* and *p* = 0.15 for *fat-4 fat-1* mutants). These results indicate that the PUFA EPA is required dynamically in adult animals for adaptation of the AWC-mediated olfactory response. Consistent with this finding, expression from the GPCR, STR-2 promoter which is down stream of EGL-4 activity [26],[52] was unchanged in either *fat-3* or *fat-4 fat-1* mutants (Table 1).

**Table 1:** *str-2* expression phenotype of adaptation and odor-induced EGL-4 translocation mutants.

Cells expressing (p)*str-2*::RFP	2 AWC^ON^	1 AWC^OFF^/1 AWC^ON^	2 AWC^OFF^	n
**Strain**				
N2 (wildtype)	0	94	6	102
Mutants				
*fat-4(wa14) fat-1(wa9)*	0	92	8	78
‡ *odr-3(n1605)*	0	100	0	150
*fat-3(wa22)*	0	96	4	110
*osm-9(ky10)*	0	92	8	73
*adp-1(ky20)*	0	93	7	70

Animals containing the transgene (*p)str-2*::RFP were scored for the *str-2* expression pattern. The chemoreceptor STR-2 is asymmetrically expressed in the AWC^ON^ and not the AWC^OFF^ cell. Data represents transgenic animals from at least three independent lines. In each case animals were scored under three categories for (*p)str-2*::RFP expression: (*p)str-2*::RFP expression in both AWC neurons; (*p)str-2*::RFP expression in only one AWC neuron; (*p)str-2*::RFP expression in neither AWC neuron.

**‡:** Data from Troemel et al. 1999 [70].

We next asked whether odor could induce nuclear accumulation of GFP::EGL-4 in the PUFA biosynthesis-defective mutants. We found that, similar to *osm-9* mutants, both *fat-3(wa22)* and *fat-4(wa14) fat-1(wa9)* mutant animals displayed normal nuclear localization of EGL-4 after prolonged odor exposure (Figure 4B – third and fourth sets of bars). Thus, even though these mutant animals accumulated GFP::EGL-4 within their nuclei, they were unable to adapt to the odor since they lacked the ability to produce PUFAs. This indicates that PUFAs are likely to act downstream of or in a parallel pathway with EGL-4 nuclear entry to promote adaptation of the AWC neuron.

To determine if PUFA signaling requires the TRPV channel OSM-9 to promote adaptation, we asked whether dietary supplementation with EPA could rescue the adaptation defects of an *osm-9(ky10)* mutant strain. When we supplemented the diets of *fat-3(wa22)*, *fat-4(wa14) fat-1(wa9)* and *osm-9(ky10)* mutants animals with EPA 24 hours prior to assaying adaptation responses, we found that EPA supplementation could rescue the butanone adaptation defect of *fat-3* and *fat-4 fat-1* mutant animals but could not rescue the adaptation defect of *osm-9* mutant animals (Figure 4E, *p* = 0.667 for odor-exposed *osm-9* populations with and without EPA treatment). This result suggests that PUFAs might signal through the TRPV channel OSM-9 to promote butanone adaptation.

Since the adaptation defects seen in PUFA biosynthesis mutants did not seem to result from a failure to accumulate EGL-4 in the AWC nucleus subsequent to prolonged odor exposure, we reasoned that PUFA signaling might, instead, function downstream of the nuclear translocation of EGL-4 to promote adaptation. To address this possibility, we asked whether supplying EPA to *egl-4* null animals would relieve their adaptation defects. Khan-Kirby *et al.*, [49] found that supplying exogenous EPA could stimulate ASH-mediated avoidance responses as well as calcium transients in an OSM-9 dependent manner. Thus, we hypothesized that exogenous EPA might be sufficient to elicit adaptation in the absence of EGL-4 if, indeed, EPA production was the only stimulus downstream of nuclear EGL-4. We found, however, that EPA administration did not rescue the adaptation defects of an *egl-4* null strain (Figure 4F). Thus, PUFA signaling is not sufficient to promote adaptation after prolonged odor-exposure in the absence of EGL-4. This may indicate either that PUFA signaling (rather than just production) is dependent on EGL-4 or that, once in the nucleus, EGL-4 promotes changes in addition to an increase in PUFAs in the AWC neuron that dampen the response to odor.

Another way to assess whether PUFA signaling might be downstream of EGL-4 nuclear entry is to ask whether loss of PUFA signaling can suppress the gain-of-function phenotype of a constitutively nuclear form of EGL-4. In Lee *et al.* [26] we describe the construction and assessment of this altered form of EGL-4. Briefly, we appended an extra nuclear localization sequence (NLS) to the N terminus of GFP within the GFP::EGL-4 fusion construct (Figure S3). This construct, designated NLS::GFP::EGL-4, was introduced into both wildtype and *egl-4(n479)* animals as a transgene. Strains expressing the NLS::GFP::EGL-4 transgene displayed GFP::EGL-4 in the AWC nuclei of both naïve and odor-exposed animals ([26] and Figure S3). The NLS::GFP::EGL-4 was fully functional, as assessed by its ability to rescue AWA-mediated chemosensory defects (EGL-4 is required in the AWA neuron for the animal's response to the odor diacetyl) [26]. AWC-mediated chemotaxis, however, was inhibited in both the wildtype and the *egl-4(n479)* genetic background (Figure 4H and [26]). One explanation consistent with our finding that nuclear EGL-4 is necessary for adaptation of the wildtype animal to odor [26] is that animals expressing a constitutively nuclear form of EGL-4 are always odor-adapted. If this is, in fact, the case, then the chemotaxis defects of the NLS::GFP::EGL-4 expressing worms should be rescued or ameliorated by removal of factors that act downstream of EGL-4 nuclear entry and not by loss of factors that are not required for adaptation. We first examined NLS::GFP::EGL-4 in an adaptation-proficient mutant background. We found that the cAMP response element binding protein (CREB) loss-of-function mutant strain, *crh-1(tz2)* [53] acts like wildtype with respect to its ability to adapt to prolonged odor exposure (Figure 4G). When we expressed NLS::GFP::EGL-4 in this mutant background, we found that these animals showed the same chemotaxis index as wildtype siblings expressing the same transgene (Figure 4H, mean C.I. for NLS::GFP::EGL-4 in wildtype = 0.17, mean C.I. for NLS::GFP::EGL-4 in *crh-1* = 0.09, *p* = 0.137). When we examined the behavior of *fat-3* mutant animals that expressed this transgene, however, we found that they showed significantly higher chemotaxis indexes than their wild-type siblings that expressed NLS::GFP::EGL-4 (Figure 4H, mean C.I. for NLS::GFP::EGL-4 in wildtype was 0.03 and the mean C.I. for NLS::GFP::EGL-4;*fat-3(wa22)* was 0.40, *p* = 0.005). In each case, the transgenic array was introduced by mating into the mutant background and wildtype siblings were used to derive the wildtype control lines. Thus, PUFAs are likely to function downstream of EGL-4's nuclear accumulation after prolonged odor exposure.

Finally, as evidence points to the TRP channel OSM-9 acting downstream of EGL-4 nuclear entry (i.e. it was not required for the nuclear translocation of EGL-4 after prolonged odor exposure (Figure 4B) but was required for adaptation of the butanone chemotaxis response (Figure 4A)), we asked whether loss of OSM-9 would suppress the NLS::GFP::EGL-4 chemotaxis defects. We found that the *osm-9(ky10)* null mutation did suppress the chemotaxis defects of the NLS::GFP::EGL-4 expressing transgenic animals (Figure 4H, mean C.I. for NLS::GFP::EGL-4 in wildtype genetic background was 0.27 and the mean C.I. for NLS::GFP::EGL-4;*osm-9(ky10)* was 0.72, *p* = 0.014). The extent of the rescue was greater than that of the *fat-3* loss-of-function in the same context. That is, *osm-9* loss rescued the C.I. back to 100% of the wildtype and *osm-9* values (first two light gray bars in Figure 4H, mean C.I. for wildtype = 0.72, mean C.I. for *osm-9* = 0.73, mean C.I. for *osm-9*;NLS::GFP::EGL-4 = 0.72, *p* = 0.818), while *fat-3* loss increased the C.I. to up to 65% of the *fat-3* alone (C.I. for wildtype alone = 0.78, C.I. for *fat-3* alone = 0.62, C.I. for *fat-3*;NLS::GFP::EGL-4 = 0.40, *p* = 0.008).

This suggests that though both OSM-9 and PUFAs are likely to function downstream of EGL-4's nuclear translocation, OSM-9 may have additional non-PUFA mediated functions downstream of nuclear EGL-4.

### Diacylglycerol signaling may function downstream of EGL-4's odor-induced nuclear entry

One event within the adaptation pathway that could occur downstream of EGL-4 nuclear entry is modulation of synaptic vesicle release. A key player in this process is diacylglycerol (DAG) [54],[55]. High DAG levels are thought to both increase the size of the readily releasable synaptic vesicle pool as well as to facilitate synaptic vesicle release via the neurotransmitter release regulator UNC-13 [54]. Likewise, dense core-vesicle release is stimulated by the DAG-activated protein kinase C (PKC) epsilon/eta, PKC-1 [56]. AWC neurons have been shown to display tonic neurotransmitter release in the absence of odor which is inhibited in the presence of odor [13]. Adaptation of the AWC neuron to an odor could result in higher rates of synaptic transmission to override the repression of synaptic release that accompanies odor signaling [13].

**Figure 5:**
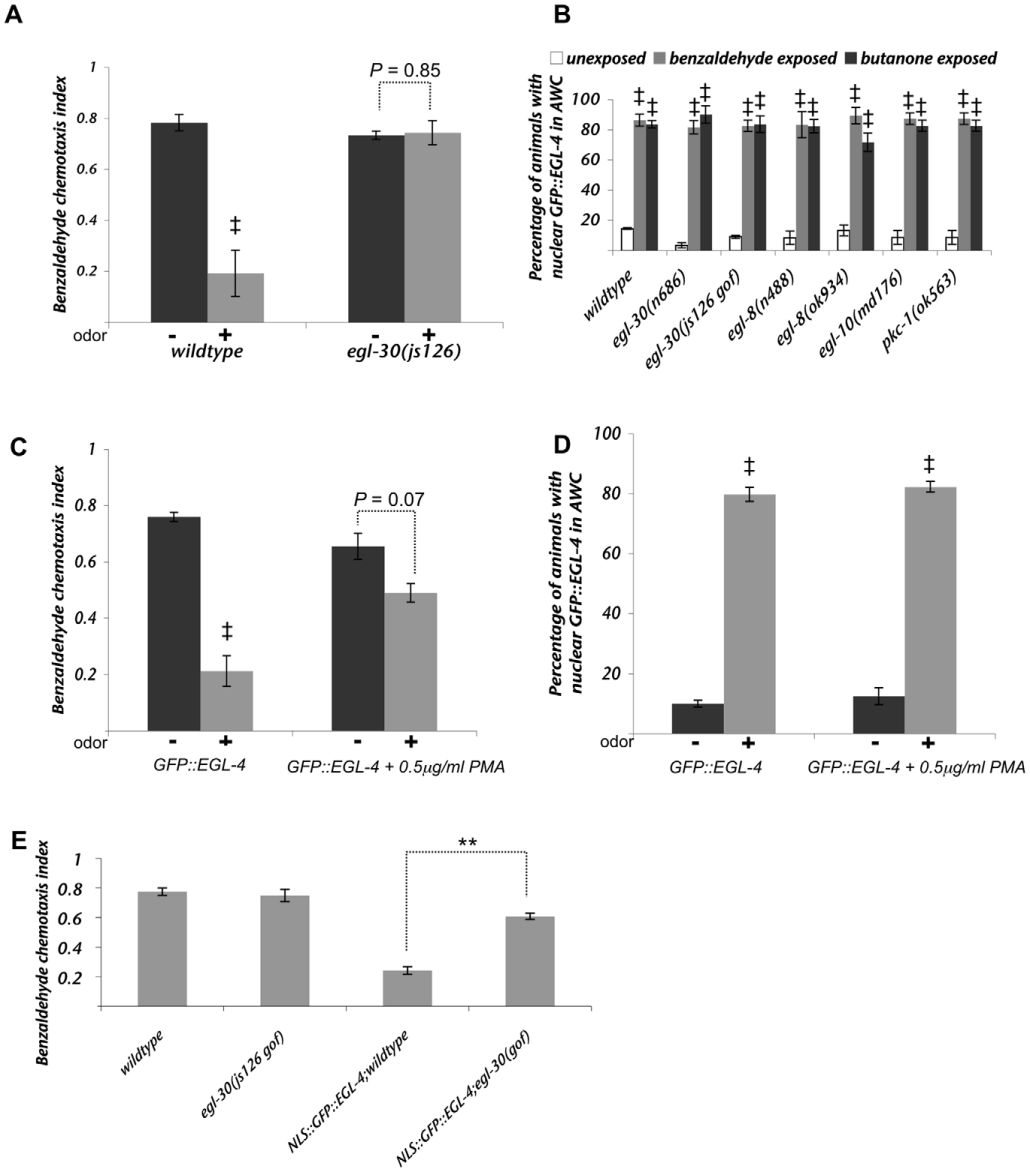
DAG signaling does not regulate the odor-induced nuclear entry of EGL-4. (A) A gain-of-function mutation in the Gα_q_ protein EGL-30 causes defects in AWC adaptation behavior as previously described by Matsuki *et al.* [4]. (B) DAG signaling mutants containing a GFP-tagged EGL-4 transgene were exposed to benzaldehyde adaptation mix for 80 minutes. The nuclear translocation of EGL-4 does not require the DAG signaling molecules EGL-30, EGL-8, EGL-10 or PKC-1. (C,D) GFP-tagged EGL-4 expressing wild-type animals (*pyIs500*) were exposed for 80 minutes to benzaldehyde adaptation mix containing the DAG analog; phorbol myristate acetate (PMA). Exposure to PMA inhibits the adaptation response at the behavioral level (C) but not the nuclear translocation of GFP::EGL-4 (D). (E) Animals with a constitutively nuclear gain-of-function allele of *egl-4* (NLS::GFP::EGL-4) are constitutively adapted. Assaying the chemotaxis behavior of this line in an *egl-30(js126)* mutant background suppressed the constitutively adapted phenotype of NLS::GFP::EGL-4 expressing animals suggesting that DAG functions downstream of EGL-4's nuclear entry. “+” bars indicate adapted animals and “−” bars denote unadapted animals. Error bars represent the S.E.M. ** indicates statistical significance at *p*<0.005. ^‡^ indicates significant difference at *p*≤0.05 between unadapted and adapted mutant bars or unadapted and adapted wildtype bars. *P* values calculated using the Student's *t*-test.

DAG levels are increased by the G_q_α protein, EGL-30 and decreased by the G_o_α GOA-1 [54]. A gain-of-function allele of *egl-30(js126)* stimulates the phospholipase C, EGL-8, to increase DAG levels [57]. This mutation blocks adaptation to benzaldehyde ([4] and Figure 5A). To determine whether DAG inhibits adaptation by blocking the nuclear translocation of EGL-4, we examined the effect of mutations in several genes that encode key DAG-signaling molecules on this process. GFP::EGL-4 localization was examined in the AWC neurons of: *egl-8(n488)* and *egl-8(ok934)*mutants, which lack the phospholipase C beta homolog and, therefore, the ability to make DAG [54],[57]; loss-of-function mutants in *egl-30(n686)* that would decrease DAG levels and *egl-30(js126)* gain-of-function mutants that would increase DAG levels; the GOA-1 specific RGS, *egl-10* mutants which should have more active GOA-1 and thus lower DAG; and in the *pkc-1(ok563)* mutants which should have lower dense core vesicle release. We found that GFP::EGL-4 nuclear accumulation was not affected by any of these mutations (Figure 5B).

To further examine the potential ability of DAG to regulate EGL-4's nuclear entry we asked whether exogenous application of the DAG analog, phorbol myristate acetate (PMA) could affect EGL-4 nuclear accumulation. As was previously described (Matsuki *et al*. [4], PMA was able to block adaptation (Figure 5C). When we examined GFP::EGL-4 expressing animals that had been incubated with both odor and PMA, we found that GFP::EGL-4 accumulated in the AWC nucleus at the same rate in these animals as the control animals that had been incubated with just buffer and odor (Figure 5D). Importantly, the same starting populations that were used in the behavioral assays were also examined microscopically for GFP::EGL-4. Thus, high DAG levels were able to block behavioral adaptation even in populations that showed nuclear EGL-4 and would have been adapted had they not been exposed to PMA. This suggests that in order for nuclear EGL-4 to promote adaptation, DAG levels might need to be regulated. It is also possible that ectopically increasing DAG levels blocked adaptation via a parallel pathway.

To test the hypothesis that DAG acts downstream of nuclear EGL-4, we asked whether the chemotaxis defects of the constitutively nuclear allele of *egl-4* (Figure S3 and [26]) could be suppressed by excess DAG. The NLS::GFP::EGL-4 was expressed in the gain-of-function *egl-30(js126)* mutant background that is proposed to have high DAG levels within the AWC neuron [4]. We found that the *egl-30(js126*) mutation did indeed suppress the chemotaxis defects of NLS::GFP::EGL-4 animals (Figure 5E, mean C.I. for NLS::GFP::EGL-4 in wildtype = 0.24 and the mean C.I. for NLS::GFP::EGL-4;*egl-30(js126)* = 0.6, *p* = 0.003). This implies that high DAG may act downstream of EGL-4's nuclear entry to block adaptation and thus, the ability of the cell, perhaps via EGL-4, to regulate DAG levels is important for appropriate down-regulation of chemotaxis in response to odor stimulation. It is also possible that the high DAG levels produced in the *egl-30* gain-of-function block adaptation by another process that is independent of, rather than down stream of nuclear EGL-4.

## Discussion

### Integration of G-protein signaling over time to promote adaptation

The mechanisms of AWC olfaction and vertebrate phototransduction appear to be quite similar. In the absence of light, photoreceptor cells exhibit an inward “dark current” of calcium through cGMP-gated channels. Upon absorbing a photon, the receptor rhodopsin becomes enzymatically active and catalyzes the activation of the G-protein, transducin, which activates a phosphodiesterase (PDE). The PDE then hydrolyzes cGMP causing the closure of the cGMP-gated channels resulting in a decrease in calcium influx [58]. Similarly, in AWC, odor has been shown to decrease calcium levels [13] probably by closing cGMP-gated channels (TAX-2 and TAX-4 - [14],[15]). It is postulated that the closing of the cyclic nucleotide gated channel results from activation of a Gα such as ODR-3, which may in turn inhibit a guanylyl cyclase or stimulate a phosphodiesterase thereby lowering cGMP levels within AWC in response to odor.

The rapid adaptation of the vertebrate visual response is primarily regulated by the dampening of the Gα by the regulator of G protein signaling RGS9 [59]. Since physiological examination of the AWC neurons is still in its infancy, we know little about the rapid events (in the timescale of seconds to minutes) that are required for odor adaptation of the AWC neurons. Using behavioral, genetic and cell biological assays though, we have been able to examine how adaptation develops over the timescale of tens of minutes to hours and we have gained some insight into the molecular nature of these changes.

After 30 minutes of exposure to odor, the attractiveness of the odor, as measured by the chemotaxis index, (CI) falls to about 75% of the initial value and, as the exposure time is lengthened, the odor's attractiveness is further diminished so that by 80 minutes of exposure, the odor may be completely ignored (the CI is close to 0). The short-term adapted state is labile in that it can be reversed after only 30 minutes of recovery in the absence of odor [26]. As exposure time is lengthened, however, the initial, easily reversed adaptation develops into an enduring form that can persist for more than three hours. The progression from short- to long-term adaptation might be attributed to the change in EGL-4's targets as it relocalizes from the cytoplasm of the naive animal into the nucleus of the 60-plus minute exposed worm. In short-term adaptation, we have evidence that EGL-4 phosphorylates the odor responsive cyclic nucleotide gated channel [3]. In general, phosphorylation is a labile modification and most sites are dephosphorylated within tens of minutes [60]. The transience of phosphorylation would allow for rapid recovery from short-term adaptation. In long-term adaptation, EGL-4 becomes concentrated in the AWC nucleus where it alters transcription of at least one G-protein coupled receptor (STR-2) [26] and probably other genes. Transcriptional changes are usually long lasting as the length of the altered state endures for as long as the newly synthesized protein products persist.

**Figure 6:**
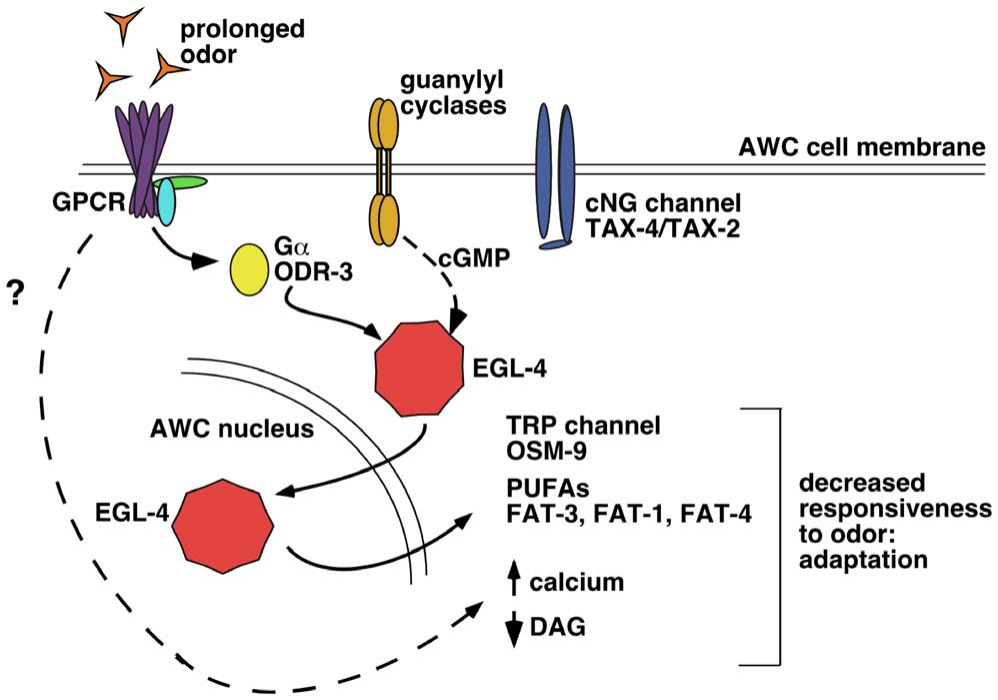
Proposed model for adaptation of the AWC neurons to prolonged odor exposure. After prolonged exposure to AWC-sensed odors, the Gα subunit protein ODR-3 triggers EGL-4 to accumulate within the nucleus of the AWC sensory neuron. This translocation depends on the ability of EGL-4 to bind cGMP, which is likely to be produced by guanylyl cyclases such as ODR-1 and DAF-11. The dashed arrow for cGMP signaling indicates that we do not understand how cGMP fluxes fit into the picture. The naïve cytosolic localization of EGL-4 depends on intact dendrite and cilia structures. Downstream of the accumulation of EGL-4 within the nucleus, fatty acids (PUFAs and DAG), calcium levels, and a transient receptor potential channel type V (OSM-9) facilitate adaptation of the chemosensory response of AWC. Our data provide a genetic model of adaptation in AWC that does not rule out the possibility of direct modulation of DAG and calcium levels as indicated by the dashed arrow and question mark.

Using odor-induced nuclear accumulation as a cell-specific biological tool to probe this one part in the process of olfactory adaptation, we examined players in the pathways that are required for generating the odor signal such as: the G-protein and its modulators; the guanylyl cyclase; the calcium channels and calcium based modulators; the PUFA pathway and finally the diacylglycerol pathway that may be involved in synaptic transmission. From this analysis, the Gα protein ODR-3 emerged as the only signal transduction molecule that was required to integrate odor signaling over time to allow adaptation of AWC-mediated olfactory responses (Figure 2B – second set of bars). Another factor that affected GFP::EGL-4 nuclear accumulation was GRK-2 (Figure 2G). This is in accord with the finding that GRK-2 is required to boost the signal from ODR-3 [35]. The role of cGMP in this process is, at present, unclear. So far, we have shown that it is required for EGL-4's ability to enter the nucleus [26] but we have not been able to demonstrate that changes in its levels affect nuclear translocation of EGL-4. Once we can actually monitor the levels of cGMP within AWC, this may be a more tractable task. The next set of factors in the proposed signaling pathway, the cGMP gated calcium channels, did not affect odor-induced GFP::EGL-4 nuclear accumulation as mutants lacking either channel subunit were able to respond to odor exposure in our paradigm (Figure 3B). Thus, we have been able to show that events downstream of Gα signaling do not affect odor's ability to induce GFP::EGL-4 nuclear accumulation. This indicates that the odor signaling pathway feeds into EGL-4-mediated adaptation at the point of, or downstream of, Gα signaling but before calcium levels change (Figure 6) and that some of the long-lasting changes could include up regulation of the PUFA pathway; increased expression of the TRP V channel, OSM-9; lowered expression of the DAG pathway and EGL-30. Though the AWC neuron and vertebrate rods share signaling properties and downstream circuitry, it is an open question as to whether long-term adaptation of the retinal cells might also involve nuclear accumulation of the rod's PKG.

### PUFA signaling downstream of adaptation of AWC

Studies by Kahn-Kirby et al. into the nociceptive neuron pair, the ASH, have provided much insight into TRP channel function within sensory neurons of *C. elegans* [49]. OSM-9 is required in conjunction with a second TRP channel subunit, OCR-2, to mediate the primary sensory response in the ASH sensory neuron but in the AWC neuron, it is required only for adaptation [1],[48],[61]. In ASH, the PUFAs directly activate the heteromeric TRP channel [49]. This work indicates that PUFAs may also activate the monomeric OSM-9 channel to allow for adaptation since the loss of either OSM-9 or FAT-3 leads to butanone adaptation defects. Interestingly, we have found that PUFAs are required for adaptation to a wider range of odors than OSM-9. That is, the *fat-3* and *fat-1 fat-4* mutants are defective for their ability to adapt to benzaldehyde while the *osm-9* mutant is able to adapt to benzaldehyde like wildtype. Loss of either PUFA signaling or OSM-9 was able to suppress the gain-of-function EGL-4 allele (NLS::GFP::EGL-4). This indicates that PUFA signaling via OSM-9 might act directly downstream of EGL-4 nuclear entry.

### Polyunsaturated fatty acid signaling and neuronal plasticity

Humans require essential PUFAs in their diet [62] to provide protection against both ischemic stroke and cardiovascular disease [63]. The mechanisms underlying the contribution of PUFAs to these medical problems are poorly understood. The nematode *C. elegans*, unlike humans, are capable of synthesizing long chain PUFAs by desaturating and elongating saturated fats obtained from its bacterial food source [50],[51].

Our findings place PUFAs downstream of EGL-4's odor-induced nuclear entry. This observation implies a potential role for PUFAs at the presynaptic site of AWC. Previous studies in *C. elegans* have shown that PUFA signaling is required for efficient synaptic transmission [64]. Lesa *et al.* [64] have shown that *fat-3* mutant animals have a reduction in the number of synaptic vesicles at release sites and exhibit a concomitant reduction in neurotransmitter release. Furthermore, Marza *et al.* [65] demonstrate that *fat-3(wa22)* mutant animals exhibit mislocalization of the presynaptic proteins synaptojanin and synaptobrevin. Synaptojanin is a phosphoinositide phosphatase that promotes the uncoating of presynaptic endocytic vesicles [66] while synaptobrevin is part of the synaptic vesicle. *C. elegans* synaptojanin mutants (*unc-26*) are defective in synaptic vesicle recycling and neurotransmitter release due to a reduction in synaptic vesicle numbers [66]. *fat-3* mutant animals phenocopy these defects demonstrating that PUFAs are required to promote endocytosis at synapses by regulating synaptojanin localization at sites of release [66]. The contribution of PUFAs to synaptic transmission is a conserved mechanism as demonstrated in studies with rats that have revealed that chronic PUFA deficiency causes a decrease in synaptic vesicle number at the presynaptic sites of dopaminergic neurons of the frontal cortex [67].

Synaptic vesicles are produced in cell bodies and transported along the axon to the nerve terminal. After fusion at the pre-synaptic plasma membrane, the synaptic vesicle proteins and lipids are rapidly endocytosed via clathrin mediated mechanisms and are reused to form new synaptic vesicles. To facilitate olfactory plasticity, perhaps PUFAs control adaptation responses in AWC by regulating synaptic vesicle recycling downstream of EGL-4's nuclear entry. The fact that the TRP channel OSM-9 is required for adaptation to two of the three odors the PUFAs are required for might indicate an odor-specific role for PUFA regulated processes perhaps in the vicinity of the synapse.

### DAG signaling may act in parallel to or downstream of EGL-4 nuclear entry

DAG may act downstream of EGL-4 nuclear entry since increasing DAG levels blocks adaptation without affecting EGL-4 nuclear accumulation (Figure 5). G_o_α/G_q_α mediated DAG signaling has been shown to increase neurotransmitter release in motor neurons [55]. Since increasing DAG levels either by application of PMA or the action of a *gain-of-function egl-30* allele blocks adaptation, it is likely that synaptic transmission needs to be down regulated (or at least tightly regulated) in order for AWC to adapt to prolonged odor exposure. Once in the nucleus, EGL-4 may down-regulate EGL-30 or another factor responsible for high DAG levels since the *gain-of-function egl-30* suppresses the constitutively nuclear EGL-4's chemotaxis defects. Indeed, odor-induced dampening of synaptic transmission would be a simple way to silence AWC's output. This interpretation, however, must be tempered by the fact that such a gain-of-function could ectopically or non-specifically suppress this phenotype.

### Calcium and DAG signaling act in opposite ways for olfactory adaptation

We have reproduced the finding that calcium is required for adaptation but we also show that this requirement is actually downstream of or in parallel to EGL-4 nuclear accumulation. Thus, calcium dependent processes must be at work in promoting adaptation but what they are remains obscure. NCS-1 was an obvious candidate [47] but we show that its loss does not affect adaptation of the AWC neuron and calcineurin acts in the opposite fashion to dampen adaptation. Indeed, increases in synaptic transmission might require higher calcium levels and could counteract the odor-induced decreases in calcium and lower synaptic transmission that accompanies odor exposure [13]. The finding that both decreased DAG and increased calcium are required downstream of or in parallel to EGL-4 nuclear accumulation indicates that we really do not understand where or how they act. Both DAG and calcium increase synaptic transmission and it is difficult to see how both could act on this one process. Thus, it is likely that each acts on a different process and that one or the other may affect synaptic transmission.

### Structural integrity of AWC cilia is critical for proper localization of EGL-4

Odor-naive wildtype and most mutant animals we examined in our candidate screen displayed diffusely cytoplasmic GFP::EGL-4 (Figure 1C and Figure S1 Chart 1 - unexposed animals). Animals with severe structural defects in the cilia and distal region of the dendrites of AWC, however, exhibited constitutively nuclear GFP::EGL-4 (Figure 1F–1J). Further, the incidence or penetrance of nuclear GFP::EGL-4 in the naive animal correlated strongly with the penetrance of the strain's structural defects (Figure 1L). Since odor exposure was able to induce significant increases in the percent of animals with nuclear GFP::EGL-4, even in the structurally defective signaling mutant strains (Figure 2B - *rgs-3* and Figure 3B - *tax-2*, *tax-4* butanone and benzaldehyde exposed bars versus unexposed bars; *p* = 0.005 for *rgs*-3 and *p* = 0.05 for *tax-2* and *tax-4*), this suggests that the mutations that cause severely deformed cilia and distal region of dendrites do not affect the dynamic translocation of EGL-4 to the nucleus.

There are several explanations for the constitutively nuclear EGL-4 phenotype of severely AWC cilia/dendrite defective mutants. One explanation is that EGL-4 may be tethered in the cytosol of the cilia/dendrite in the AWC by association with other proteins. These tethering proteins may be sensitive to cilia morphology and be unable to correctly retain EGL-4 in the cytosol when this region of the cell is deformed. This tether model suggests that EGL-4 may have a propensity to enter the nucleus and that dissociation of the link between certain tethering proteins and EGL-4 may be a key step in the translocation of EGL-4. Indeed, as Mukhopadhyay *et al.* [33] showed, sensory signaling is required to maintain wildtype cilia morphology. In this work, Mukhopadhyay *et al.* demonstrated that continual signaling by molecules including TAX-2 and TAX-4 is required to maintain proper AWB cilia architecture. In *tax-2* and *tax-4* mutants Mukhopadhyay *et al.*, [33] observed severe cilia defects in the AWB neuron. Similarly, our data show that both TAX-2 and TAX-4 are required to maintain appropriate AWC cilia structure (Figure 1I and 1J) and in these mutant strains, EGL-4 is constitutively nuclear in each cilia defective animal (Figure 1L). Another, not mutually exclusive, explanation is that, in keeping with observations by Mukhopadhyay *et al.*, perhaps baseline and continuous sensory signaling in AWC is required to both maintain cilia structure and also to keep EGL-4 in the cytosol. Thus, a certain level of continuous sensory signaling may be responsible for the localization of EGL-4 in a naive animal.

The reason that we failed to see aberrant nuclear localization of GFP::EGL-4 in the signaling-impaired naive *odr-3(n2150)* mutant strain might be because there is just enough signaling in these animals to preserve both cilia structure and normal localization of GFP::EGL-4 (see Figure S4). In fact, the *odr-3(n2150)* allele that we examined was not significantly different from the wildtype strain in its ability to respond to butanone [11]. This is in contrast to the *odr-3(1605)* allele that was significantly defective for chemotaxis to this odor and showed very severe cilia defects in AWC [11]. Though *odr-3(n2150)* showed a decrease in cilia size, as determined both by electron microscopic analysis [9] and our observations (Figure S4), the defects we observed were far less pronounced than those seen in *rgs-3*, *tax-2* or *tax-4* mutant backgrounds. Thus, we were able to dissociate the role for the Gα ODR-3 in promoting intact cilia from its role in integrating prolonged odor signaling by using the less severe *odr-3(n2150)* allele.

We have also examined the localization of EGL-4 in other neurons and the AWCs are the only cells that we could identify in which EGL-4 is not constitutively nuclear (unpublished observations by Jeff Eastham-Anderson and N.D.L). Furthermore, EGL-4's ability to accumulate within the AWC nucleus seems to be specific for EGL-4 as other GFP-tagged proteins that are both cytosolic and nuclear do not translocate to the nucleus after odor exposure (unpublished observations by Bi-Tzen Juang and N.D.L).

### Specificity of adaptation response versus a lack of specificity in nuclear accumulation of EGL-4

One interesting aspect of odor adaptation is its specificity. In the starved animal, adaptation to the AWC-sensed odor benzaldehyde does not affect isoamyl alcohol chemotaxis and vice versa [1]. It is intriguing to contemplate how, in the wildtype worm, specificity is maintained in light of our finding that the adaptation process involves nuclear translocation of a kinase that can promote adaptation to each odor we use and that occurs in response to each odor we studied (Figure 4H and [26]). In general, there are two possibilities: 1) EGL-4 may send a generic signal to adapt the neuron and this signal enhances or interacts with a second odor-specific signal; or 2) each odor may alter EGL-4 in such a way that nuclear EGL-4 produces an odor-specific signal that adapts the neuron to that odor.

Since we can force adaptation to occur even in the absence of odor by expressing a constitutively nuclear form of EGL-4 (Figure 4H), it suggests that an odor signal is not required for adaptation once EGL-4 is in the nucleus. However, when EGL-4 is forced to be constitutively nuclear by this manipulation, the worm's response to all odors is lost (data not shown). To reconcile these observations, we postulate that when the wildtype worm naturally adapts to one odor, this process differs in a way that allows odor to limit or shape the output of EGL-4. This could be achieved if the native form of EGL-4 enters the nucleus and there produces a signal that could cooperate with odor to promote adaptation. Alternately, once in the nucleus the native EGL-4 might induce gene expression of adaptation machinery that is odor-specific.

Our evidence is that there are parts of the adaptation machinery that act down stream of EGL-4's nuclear entry that are required for adaptation to specific odors, (OSM-9) and that there are parts that are required for all odors we have tested (PUFAs). Since loss of *osm-9* renders animals defective for adaptation to butanone but leaves their ability to adapt to benzaldehyde intact [1], it will be interesting to understand whether any of the signals that EGL-4 produces when it is in the nucleus are odor-specific or whether odor in conjunction with a generic EGL-4 signal promotes odor-specific adaptation.

## Materials and Methods

### Strains and maintenance

Bristol N2, *odr-3(n2150)*, *egl-4(n479)*, *gpb-2(pk751)*, *gpb-2(sa603)*, *gpc-1(pk298)*, *fat-3(wa22*), *fat-4(wa14) fat-1(wa9*), *rgs-3(vs19)*, *egl-30(js126)*, *crh-1(tz2)*, *tax-2(p671)*, *tax-4(p678)*, *osm-9(ky10)* strain JZ1032 (made by out-crossing CX10 one extra time prior to assaying), *pyIs500[(p)odr-3::GFP::EGL-4*; *(p)odr-1::RFP*; *(p)ofm-1::GFP] him-5*, *gpb-2(sa603);him-5 pyIs500*, *gpb-2(pk751);him-5 pyIs500*, *gpc-1(pk298);him-5 pyIs500*, *unc-18(md299);him-5 pyIs500*, *mbk-1(pk1389);him-5 pyIs500*, *daf-2(e1370);him-5 pyIs500*, *daf-3(e1376);him-5 pyIs500*, *let-60(n1046);him-5 pyIs500*, *let-23(sa62);him-5 pyIs500*, *gpa-2(pk16);Ex[pyIs500]*, *arr-1(ok401);him-5 pyIs500*, *rgs-3(vs19);him-5 pyIs500*, *gcy-31(ok296);him-5 pyIs500*, *gcy-36(db42);him-5 pyIs500*, *grk-2(rt97);him-5 pyIs500*, *tax-4(pr678);him-5 pyIs500*, *tax-2(p671);him-5 pyIs500*, *cng-1(jh111);him-5 pyIs500*, *cng-3(jh113);him-5 pyIs500*, *ncs-1(qa406);him-5 pyIs500*, *unc-2(e55);him-5 pyIs500*, *tax-6(jh107);him-5 pyIs500*, *tax-6(p675);him-5 pyIs500*, *ttx-3(ks5);him-5 pyIs500*, *pkc-1(ok563);him-5 pyIs500*, *egl-30(n686);him-5 pyIs500*, *itr-1(sa73);him-5 pyIs500*, *egl-8(n488);him-5 pyIs500*, *egl-8(ok934);him-5 pyIs500*, *egl-10(md176);him-5 pyIs500*, *egl-30(js126);him-5 pyIs500*, *fat-3(wa22);him-5 pyIs500*, *osm-9(ky10);him-5 pyIs500*, *fat-4(wa14) fat-1(wa9);Ex[pyIs500]*, *sel-12(ar131);him-5 pyIs500*, *che-2(e1033);him-5 pyIs500*, *che-11(e1810);him-5 pyIs500*, *pyIs500 him-5*; *Ex[(p)odr-1::ODR-1*; *elt-2::GFP]*, *odr-3(n2150);Ex[pyIs500]*, *adp-1(ky20)* is a dominant mutation (Colbert and Bargmann, [1]) and was assayed as a *cis*-heterozygote in Figure S1 as *adp-1(ky20)*; *him-5 pyIs500*/+++, N2;Ex[(p)str-2::RFP; (p)ofm-1::GFP], *fat-4(wa14) fat-1(wa9);Ex[(p)str-2::RFP*; *(p)ofm-1::GFP]*, *fat-3(wa22);Ex[(p)str-2::RFP*; *(p)ofm-1::GFP]*, *osm-9(ky10);Ex[(p)str-2::RFP*; *(p)ofm-1::GFP]*, *adp-1(ky20);Ex[(p)str-2::RFP*; *(p)ofm-1::GFP*, *pyIs500*; *Ex[(p)odr-3::ODR-3(30ng/µl)*, *(p)unc-25::RFP]*, *fat-3(wa22)*; *Ex[(p)ceh-36::FAT-3::GFP*, *(p)ofm-1::GFP]*, *N2*; *Ex[(p)odr-3::NLS::GFP::EGL-4]*, *crh-1(tz2)*; *Ex[(p)odr-3::NLS::GFP::EGL-4]*, *osm-9(ky10)*; *Ex[(p)odr-3::NLS::GFP::EGL-4]*, *fat-3(wa22)*; *Ex[(p)odr-3::NLS::GFP::EGL-4]*, *egl-30(js126)*; *Ex[(p)odr-3::NLS::GFP::EGL-4].* NGM plates were seeded with *E. coli* strain OP50 and maintained according to standard protocol [68]. Hermaphrodite mutants were crossed into *pyIs500* males and F1 cross progeny picked by identifying transgene-containing (green) worms. F2 animals were picked and genotyped by behavioral analysis, PCR, visual phenotypes or sequencing and then homozygosed for the mutation of interest and the *pyIs500* transgene.

### Chemotaxis and adaptation assays

Chemotaxis was performed as described previously [1],[8],[9]. Briefly, 10mls of 1.6% agar in assay buffer was placed into a 10cm petridish. One microliter of odor diluted into ethanol and one microliter of 100mM sodium azide (an anesthetic) was placed at opposite ends of the hardened agar lining the petridish. Worms were washed from their growth plates into S- Basal in 1.5 ml microcentrifuge tubes and collected by nanofuge centrifugation for 6 seconds this was repeated twice before placing concentrated animals at the origin which was equidistant from either odor or ethanol spots. Chemotaxis assays were initiated by wicking the liquid from the animals. The chemotaxis index of each population was determined by counting the number of animals at the odor and subtracting from that the number at ethanol and diving this number by the total number of animals that had left the origin. Odors were diluted as follows unless otherwise stated: 1µl benzaldehyde (Sigma) in 200µl EtOH, 1µl butanone (Sigma) in 1000 µl EtOH, 1µl isoamyl alcohol (Sigma) in 1000µl EtOH and 1µl 2,3 pentanedione (Sigma) in 10000 µl of EtOH. Odor adaptations were performed as described elsewhere [1],[3],[8]. Briefly, animals were treated as described for a chemotaxis assay but instead of being placed onto the petridish, the liquid in the microcentrifuge tube was replaced with either S-Basal buffer alone or S-Basal and odor. Odors were diluted as follows; 9µl benzaldehyde into 100ml S-Basal, 12µl butanone into 100ml S-Basal buffer, 1µl isoamyl alcohol into 100ml S-Basal and populations of animals were exposed to the diluted odor for 80 minutes during long-term exposure. Plate adaptation assays for isoamyl alcohol were performed as described previously [1]. Odor exposures for 60 minutes were carried out for JZ1032 (*osm-9(ky10*)) (Figure 4A and 4E) and *egl-30(js126)* animals (Figure 5A). For the mutants *fat-4 fat-1*, *fat-3*, *fat-3;NLS::GFP::EGL-4* which all exhibited uncoordinated (*unc*) phenotypes, modified adaptation assays were performed (Figure 4) by placing worms at the center of the assay plate. By modifying the origin point, shorter taxis distances were traveled to overcome the *unc* phenotype of these animals (Figure S5). For all assays between 100 and 200 animals were assayed in each assay and each assay was repeated on separate days between three and five times.

### EGL-4 nuclear accumulation assays

Four to five L4 animals were picked onto a 9cm OP50 seeded plate and incubated at 25°C. Animals were washed from these plates and accumulation assays were performed by exposing animals containing an integrated copy of *(p)odr-3::GFP::EGL-4;(p)ofm-1::GFP;(p)odr-1::RFP (*strain name *pyIs500)* to odor in S-Basal (pre-exposed) or S-Basal (un-exposed) for 80 minutes and then scoring the number of worms exhibiting GFP in one AWC nucleus after butanone exposure or in both AWC nuclei after benzaldehyde or isoamyl alcohol exposure under 40× magnification. Wildtype *pyIs500* worms were included for every translocation assay as a positive control using ≥0.75 percentile as the baseline for successful control assays and treatment assay inclusion. Between twenty and fifty animals were scored for each translocation assay and repeated on separate days three to five times.

### PMA, EGTA, and 8-br-cGMP treatments

Wildtype *pyIs500* animals were washed three times with ddH_2_O and soaked in buffer plus benzaldehyde (9µl of benzaldehyde into 100mls S-Basal) with 0.5µg/ml PMA (Sigma) for 80 minutes. After this pre-exposure, animals were washed three times with ddH_2_O and tested for chemotaxis and the subcellular localization of GFP::EGL-4 in AWC. For EGTA treatment, wildtype *pyIs500* animals were pre-incubated in 50mM EGTA (pH 7.0 in water) for 2 hours before standard benzaldehyde exposure in S-Basal. Benzaldehyde exposed animals were then assayed for chemotaxis and examined for their subcellular localization of GFP::EGL-4 in AWC, as described. For the preparation of 8-br-cGMP (Sigma) containing plates, 5mM final concentration of 8-br-cGMP was prepared as described by van der Linden et al. [69]. The 8-br-cGMP plates were then seeded with *E. coli* OP50 24 hrs later. Four to five L4 animals were picked to these plates and their progeny assayed. Naïve and odor exposed worms were examined for the localization of GFP::EGL-4 (Figure S1 - 8-br-cGMP plate). For 8-br-cGMP soaking, animals were soaked in 100 mM solution of 8-br-cGMP in S-Basal for 1 hr and exposed to odor for 80 mins (Figure S1 - 8-br-cGMP soak). Unexposed animals were examined in parallel with S-Basal instead of benzaldehyde containing adaptation mix after EGTA, PMA and 8-br-cGMP treatments.

### Plasmid construction and transgenic strains

*(p)odr-3::GFP::EGL-4* was constructed from *(p)egl-4::GFP::EGL-4* (a generous gift from M. Fujiwara) by placing GFP sequences and the first few coding sequences of EGL-4 downstream of *(p)odr-3. (p)odr-3::GFP::EGL-4* was co-injected (50 ng/µl) with the AWC marker *(p)odr-1::RFP* (25 ng/µl) and coelomocyte marker *(p)ofm-1::GFP* (25 ng/µl) into N2 animals, and the subsequent transgenic line was integrated by TMP method to form the integrated strain *pyIs500.* The *egl-4* gain-of-function allele was made by inserting an extra NLS coding sequence to the 5′ end of GFP coding sequences in the *(p)odr-3::GFP::EGL-4* construct. This was accomplished by site directed mutagenesis. The resulting construct was called *(p)odr-3*::NLS::GFP::EGL-4. ODR-3 overexpression lines were generated using *(p)odr-3::ODR-3* (a kind gift from Denise Ferkey). ODR-1 overexpression lines were generated using (p)*odr-1*::ODR-1 (injected at 1µg/µl). The (*p*)AWC::FAT-3::GFP rescue construct was made by sub-cloning cDNA into an AWC specific promoter construct, *(p)ceh-36::GFP* (a kind gift from John F. Etchberger and Oliver Hobert) at the N terminus of GFP by using *Kpn*I and *Msc*I sites. The *ceh-36* promoter was truncated by John F. Etchberger and Oliver Hobert until AWC-specific expression was achieved.

### Polyunsaturated fatty acid dietary supplementation

Eicosapentaenoic acid (EPA) stocks were prepared by diluting EPA fatty acid salt (Nu-Chek Prep, Elysian, MN) to 100 mM in ddH_2_0 immediately prior to making plates as described by Watts *et al.* [50] and Kahn-Kirby *et al.* [49]. NGM solution was prepared with the addition of 0.1% tergitol (NP-40, Sigma). Once the agar was cooled to 45°C–50°C, lipids were added slowly, with stirring, to a final concentration of 160µM then dried at room temperature for at least 24 hours and seeded with OP50 *E. coli*.

## Supporting Information

Figure S1 Mutant animals for a variety of signaling pathways were assayed for the ability of EGL-4 to translocate to the nucleus after prolonged odor exposure. Error bars represent S.E.M.
(0.90 MB TIF)

Figure S2 Overexpression of ODR-1 causes developmental and adaptation defects in AWC ([38]; [7] and N.D.L.). (A) The chemotaxis response of three transgenic lines of ODR-1 overexpressing animals to 2,3 pentanedione was examined. The odor 2,3-pentanedione is sensed by the AWC OFF cell. (B) The adaptation behavior of three transgenic lines overexpressing ODR-1 was examined to the AWC sensed odor butanone. Error bars represent S.E.M. ** Indicates p less than 0.005 and * indicates p less than 0.05 significant differences.
(0.25 MB TIF)

Figure S3 The authors have previously demonstrated that appending an extra nuclear localization sequence (NLS) onto the N terminus of EGL-4 to make a (p)odr-3::NLS::GFP::EGL-4 expressing line was sufficient to constitutively force EGL-4 into the nucleus of AWC [26]. Consequently the animals displayed a constitutively adapted phenotype at the behavioral level. This fluorescent confocal image shows a naïve NLS::GFP::EGL-4 expressing animal with EGL-4 in the nucleus of AWC.
(0.77 MB TIF)

Figure S4 Fluorescent confocal image of the AWC neuron of an *odr-3(n2150)* mutant animal. Structural defects of *odr-3(n2150)* are juxtaposed with representative images of wild-type and *rgs-3(vs19)* for comparison. The white dotted box highlights the defect. The *n2150* mutant fails to form wild-type fan shaped cilia.
(0.45 MB TIF)

Figure S5 Representation of standard and modified assay plates. The mutant animals *fat-4(wa14)fat-1(wa9)* and *fat-3(wa22)*, which all exhibited uncoordinated (*unc*) phenotypes, were assayed using modified adaptation assays by placing worms at the center of the assay plate. By modifying the origin point, shorter distances were traveled to overcome the *unc* phenotype problem with assaying these animals. Modified assays were also used to assay *fat-3(wa22)*; Ex [(p)*odr-3*::NLS::GFP::EGL-4] animals and *fat-3(wa22)*; Ex[(p)AWC::FAT-3] animals.
(0.23 MB TIF)

Table S1 List of mutant backgrounds tested for the ability to regulate EGL-4's entry to the nuclei of AWC after prolonged odor exposure. [71] Patterson GI, Koweek A, Wong A, Liu Y, Ruvkun G (1997) The DAF-3 Smad protein antagonizes TGF-beta-related receptor signaling in the Caenorhabditis elegans dauer pathway. Genes Dev 11: 2679–2690. [72] Antebi A, Culotti JG, Hedgecock EM (1998) daf-12 regulates developmental age and the dauer alternative in Caenorhabditis elegans. Development 125: 1191–1205. [73] Zwaal RR, Mendel JE, Sternberg PW, Plasterk RH (1997) Two neuronal G proteins are involved in chemosensation of the Caenorhabditis elegans Dauer-inducing pheromone. Genetics 145: 715–727. [74] Morris JZ, Tissenbaum HA, Ruvkun G (1996) A phosphatidylinositol-3-OH kinase family member regulating longevity and diapause in Caenorhabditis elegans. Nature 382: 536–539. [75] Murphy CT, McCarroll SA, Bargmann CI, Fraser A, Kamath RS, et al. (2003) Genes that act downstream of DAF-16 to influence the lifespan of Caenorhabditis elegans. Nature 424: 277–283. [76] Hirotsu T, Saeki S, Yamamoto M, Iino Y (2000) The Ras-MAPK pathway is important for olfaction in Caenorhabditis elegans. Nature 404: 289–293. [77] Aroian RV, Koga M, Mendel JE, Ohshima Y, Sternberg PW (1990) The let-23 gene necessary for Caenorhabditis elegans vulval induction encodes a tyrosine kinase of the EGF receptor subfamily. Nature 348: 693–699. [78] Dal Santo P, Logan MA, Chisholm AD, Jorgensen EM (1999) The inositol trisphosphate receptor regulates a 50-second behavioral rhythm in C. elegans. Cell 98: 757–767. [79] Raich WB, Moorman C, Lacefield CO, Lehrer J, Bartsch D, et al. (2003) Characterization of Caenorhabditis elegans homologs of the Down syndrome candidate gene DYRK1A. Genetics 163: 571–580. [80] Hobert O, Mori I, Yamashita Y, Honda H, Ohshima Y, et al. (1997) Regulation of interneuron function in the C. elegans thermoregulatory pathway by the ttx-3 LIM homeobox gene. Neuron 19: 345–357. [81] Hata Y, Slaughter CA, Sudhof TC (1993) Synaptic vesicle fusion complex contains unc-18 homologue bound to syntaxin. Nature 366: 347–351. [82] Cheung BH, Arellano-Carbajal F, Rybicki I, de Bono M (2004) Soluble guanylate cyclases act in neurons exposed to the body fluid to promote C. elegans aggregation behavior. Curr Biol 14: 1105–1111. [83] Hukema RK, Rademakers S, Dekkers MP, Burghoorn J, Jansen G (2006) Antagonistic sensory cues generate gustatory plasticity in Caenorhabditis elegans. EMBO J 25: 312–322. [84] Kitagawa N, Shimohama S, Oeda T, Uemura K, Kohno R, et al. (2003) The role of the presenilin-1 homologue gene sel-12 of Caenorhabditis elegans in apoptotic activities. J Biol Chem 278: 12130–12134.
(1.22 MB PDF)
