# Supplementary figures and images for: Regulators of AWC-Mediated Olfactory Plasticity in Caenorhabditis elegans
Source: PLoS Genet. 2009 Dec 11;5(12):e1000761. doi: 10.1371/journal.pgen.1000761 (PMC2780698; doi:10.1371/journal.pgen.1000761)

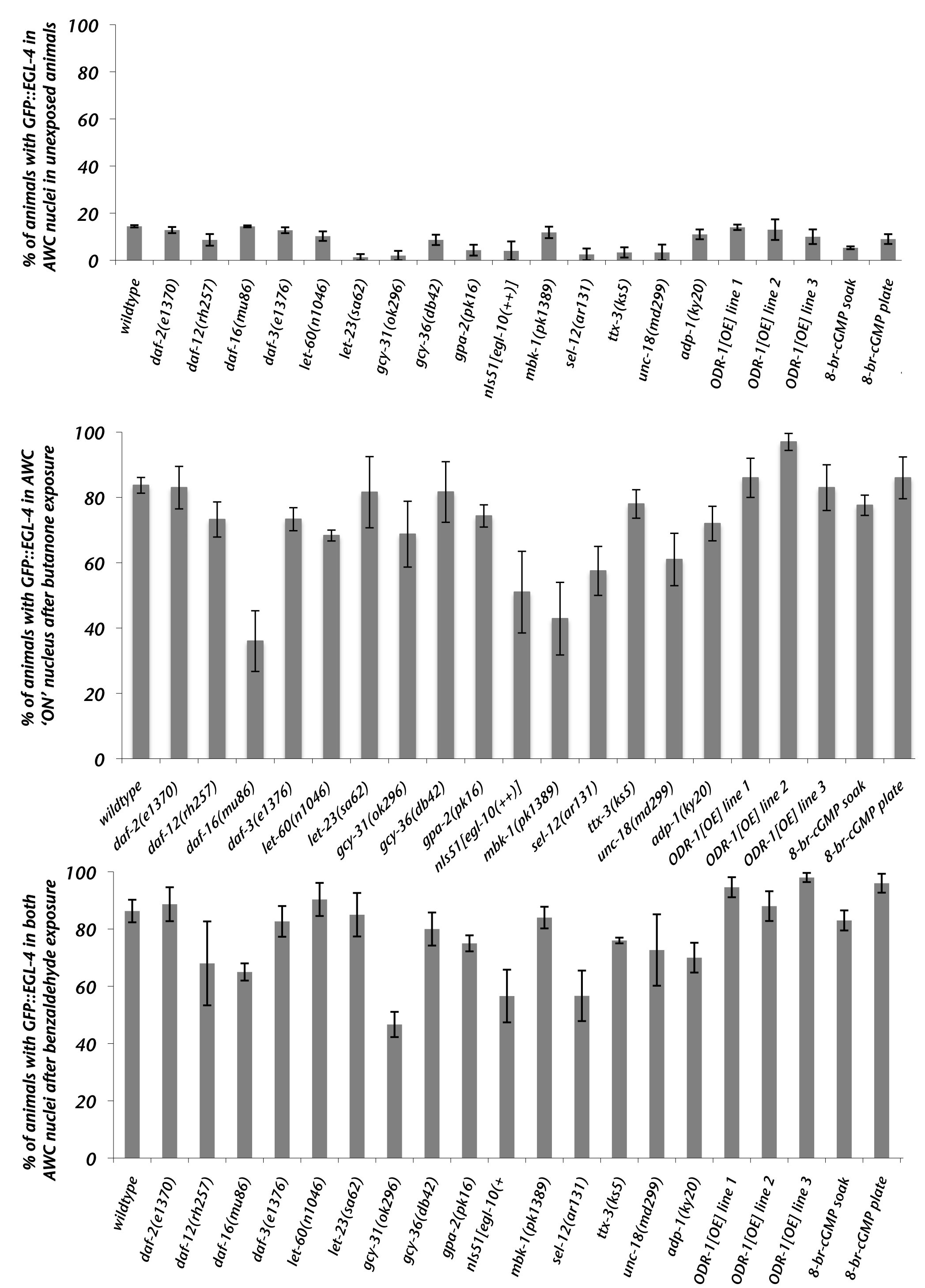

Supplement: Figure S1 — Mutant animals for a variety of signaling pathways were assayed for the ability of EGL-4 to translocate to the nucleus after prolonged odor exposure. Error bars represent S.E.M. (0.90 MB TIF) [file pgen.1000761.s001.tif]

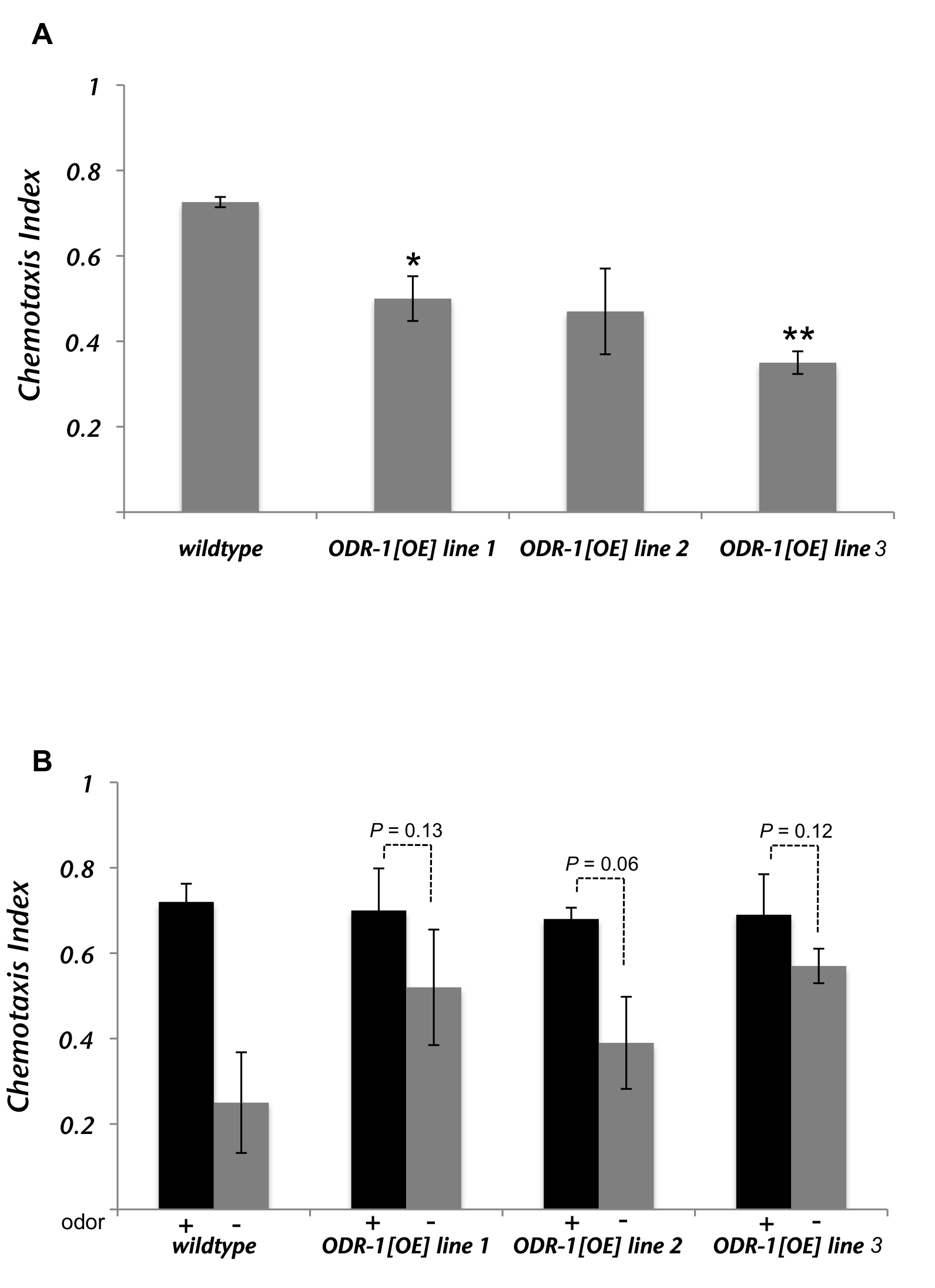

Supplement: Figure S2 — Overexpression of ODR-1 causes developmental and adaptation defects in AWC ([38]; [7] and N.D.L.). (A) The chemotaxis response of three transgenic lines of ODR-1 overexpressing animals to 2,3 pentanedione was examined. The odor 2,3-pentanedione is sensed by the AWC OFF cell. (B) The adaptation behavior of three transgenic lines overexpressing ODR-1 was examined to the AWC sensed odor butanone. Error bars represent S.E.M. ** Indicates p less than 0.005 and * indicates p less than 0.05 significant differences. (0.25 MB TIF) [file pgen.1000761.s002.tif]

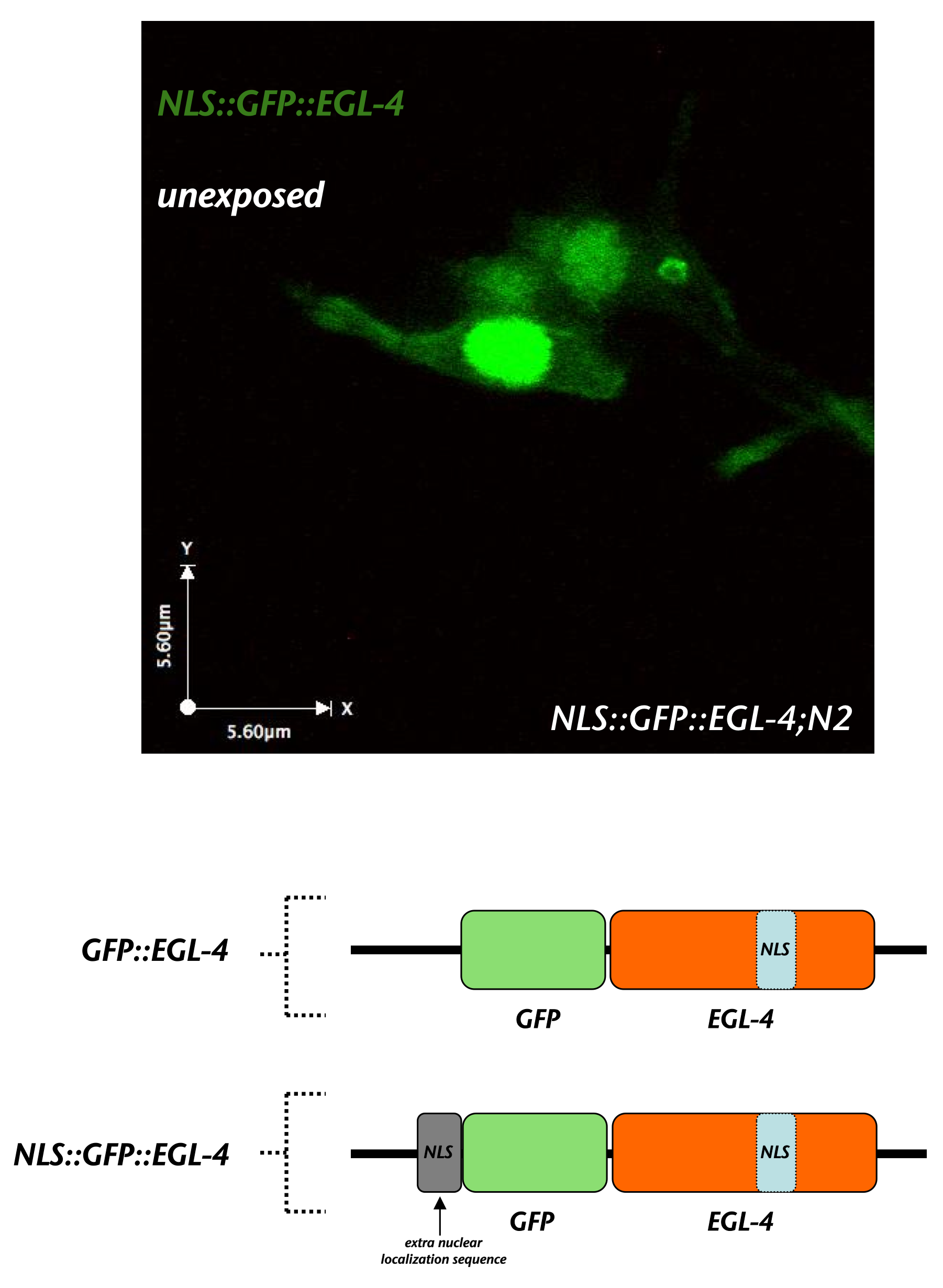

Supplement: Figure S3 — The authors have previously demonstrated that appending an extra nuclear localization sequence (NLS) onto the N terminus of EGL-4 to make a (p)odr-3::NLS::GFP::EGL-4 expressing line was sufficient to constitutively force EGL-4 into the nucleus of AWC [26]. Consequently the animals displayed a constitutively adapted phenotype at the behavioral level. This fluorescent confocal image shows a naïve NLS::GFP::EGL-4 expressing animal with EGL-4 in the nucleus of AWC. (0.77 MB TIF) [file pgen.1000761.s003.tif]

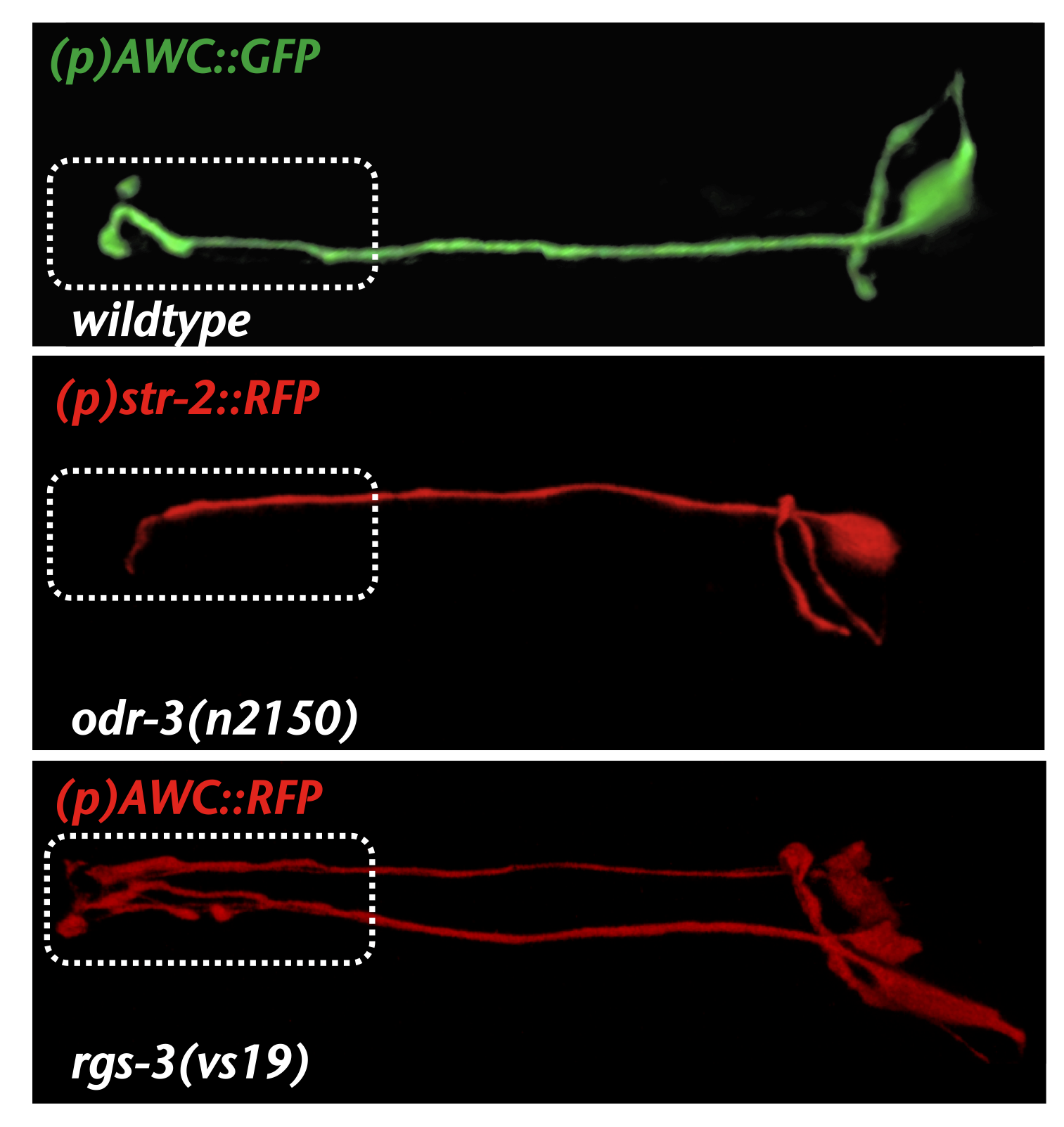

Supplement: Figure S4 — Fluorescent confocal image of the AWC neuron of an odr-3(n2150) mutant animal. Structural defects of odr-3(n2150) are juxtaposed with representative images of wild-type and rgs-3(vs19) for comparison. The white dotted box highlights the defect. The n2150 mutant fails to form wild-type fan shaped cilia. (0.45 MB TIF) [file pgen.1000761.s004.tif]

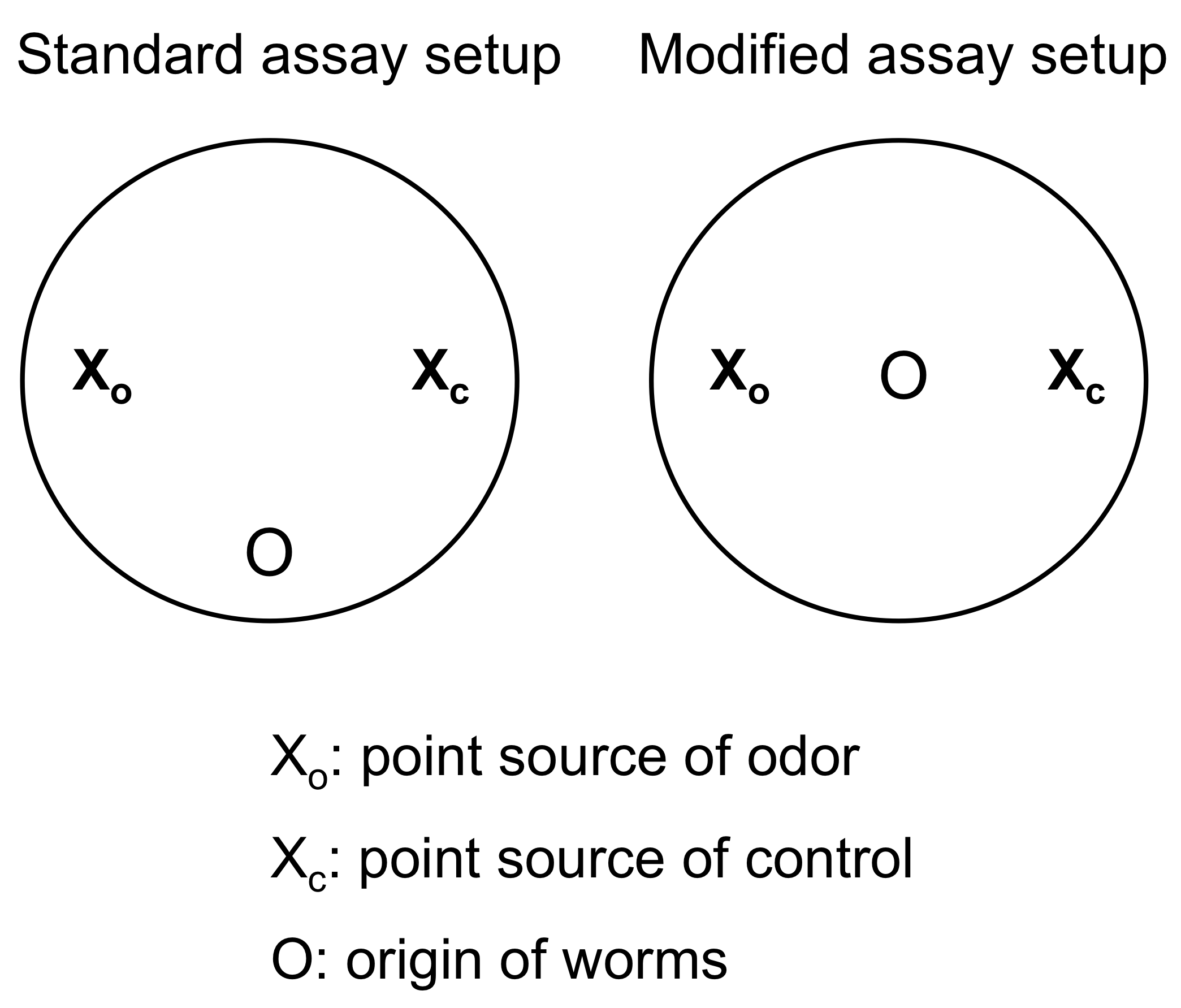

Supplement: Figure S5 — Representation of standard and modified assay plates. The mutant animals fat-4(wa14)fat-1(wa9) and fat-3(wa22), which all exhibited uncoordinated (unc) phenotypes, were assayed using modified adaptation assays by placing worms at the center of the assay plate. By modifying the origin point, shorter distances were traveled to overcome the unc phenotype problem with assaying these animals. Modified assays were also used to assay fat-3(wa22); Ex [(p)odr-3::NLS::GFP::EGL-4] animals and fat-3(wa22); Ex[(p)AWC::FAT-3] animals. (0.23 MB TIF) [file pgen.1000761.s005.tif]
