# Supplementary material for: Regulators of AWC-Mediated Olfactory Plasticity in Caenorhabditis elegans
Source: PLoS Genet. 2009 Dec 11;5(12):e1000761. doi: 10.1371/journal.pgen.1000761 (PMC2780698; doi:10.1371/journal.pgen.1000761)
Supplement: Table S1 — List of mutant backgrounds tested for the ability to regulate EGL-4's entry to the nuclei of AWC after prolonged odor exposure. [71] Patterson GI, Koweek A, Wong A, Liu Y, Ruvkun G (1997) The DAF-3 Smad protein antagonizes TGF-beta-related receptor signaling in the Caenorhabditis elegans dauer pathway. Genes Dev 11: 2679–2690. [72] Antebi A, Culotti JG, Hedgecock EM (1998) daf-12 regulates developmental age and the dauer alternative in Caenorhabditis elegans. Development 125: 1191–1205. [73] Zwaal RR, Mendel JE, Sternberg PW, Plasterk RH (1997) Two neuronal G proteins are involved in chemosensation of the Caenorhabditis elegans Dauer-inducing pheromone. Genetics 145: 715–727. [74] Morris JZ, Tissenbaum HA, Ruvkun G (1996) A phosphatidylinositol-3-OH kinase family member regulating longevity and diapause in Caenorhabditis elegans. Nature 382: 536–539. [75] Murphy CT, McCarroll SA, Bargmann CI, Fraser A, Kamath RS, et al. (2003) Genes that act downstream of DAF-16 to influence the lifespan of Caenorhabditis elegans. Nature 424: 277–283. [76] Hirotsu T, Saeki S, Yamamoto M, Iino Y (2000) The Ras-MAPK pathway is important for olfaction in Caenorhabditis elegans. Nature 404: 289–293. [77] Aroian RV, Koga M, Mendel JE, Ohshima Y, Sternberg PW (1990) The let-23 gene necessary for Caenorhabditis elegans vulval induction encodes a tyrosine kinase of the EGF receptor subfamily. Nature 348: 693–699. [78] Dal Santo P, Logan MA, Chisholm AD, Jorgensen EM (1999) The inositol trisphosphate receptor regulates a 50-second behavioral rhythm in C. elegans. Cell 98: 757–767. [79] Raich WB, Moorman C, Lacefield CO, Lehrer J, Bartsch D, et al. (2003) Characterization of Caenorhabditis elegans homologs of the Down syndrome candidate gene DYRK1A. Genetics 163: 571–580. [80] Hobert O, Mori I, Yamashita Y, Honda H, Ohshima Y, et al. (1997) Regulation of interneuron function in the C. elegans thermoregulatory pathway by the ttx-3 LIM homeobox gene. Neuron 19: 345–357. [81] Hata Y, Slaughter C [file pgen.1000761.s006.pdf]

Table S1. Overview of genetic backgrounds tested for EGL-4::GFP localization

**Gene (allele)**	**Protein**	**Function**	**Reference**
daf-2(e1370)	Insulin/IGF receptor	Diapause and longevity	Morris *et al.,* [74]
daf-12(rh257)	Hormone receptor	Dauer formation	Antebi *et al.,* [72]
daf-16(mu86)	Transcription factor	Life span	Murphy *et al.,* [75]
daf-3(e1376)	co-SMAD protein	Dauer formation	Patterson *et al.,* [71]
let-60(n1046)	GTP binding RAS proto-oncogene	Olfactory adaptation and odortaxis	Hirotsu *et al*., [23,76]
let-23(sa62)	EGF-receptor family tyrosine kinase	Vulval induction	Aroian *et al.,* [77]
cng-1(jh111)	cNG channel alpha subunit	Brood size	Cho *et al*., [42]
cng-3(jh113)	cNG channel alpha subunit	Thermotolerance	Cho *et al*., [43]
tax-4(p678)	cNG channel alpha subunit	Odortaxis and thermotaxis	Komatsu *et al*., [15]
tax-2(p671)	cNG channel beta subunit	Odortaxis and thermotaxis	Coburn & Bargmann, [14]
itr-1(sa73)	Inositol (1,4,5) triphosphate receptor	Defecation rhythm	Dal Santo *et al*., [78]
tax-6(p675)	Calcineurin A	Thermosensation and olfactory adaptation	Kuhara *et al*., [21]
unc-2(e55)	Calcium channel alpha subunit	Neurotransmitter adaptation	Schafer & Kenyon, [45]
ncs-1(qa406)	Neuronal calcium sensor	Isothermal tracking	Gomez *et al*., [47]
rgs-3(vs19)	Regulator of G protein signaling	ASH avoidance and odortaxis	Ferkey *et al*., [32]
arr-1(ok401)	Beta-arrestin	Olfactory adaptation	Palmitessa *et al*., [5]
ODR-1	Membrane guanylyl cyclase	Odortaxis	L'Etoile & Bargmann, [16]
gcy-31(ok296)	Soluble guanylyl cyclase	Oxygen sensation	Cheung *et al*., [82]
gcy-36(db42)	Soluble guanylyl cyclase	Oxygen sensation	Cheung *et al.,* [82]
odr-3(n2150)	G protein alpha subunit	Nociception and odortaxis	Roayaie *et al.,* [11]
gpc-1(pk298)	G protein gamma subunit	Gustatory plasticity	Hukema *et al*., [83]
gpb-2(sa603)	G protein beta subunit	Locomotion	Van der Linden *et al*., [36]
grk-2(rt97)	G protein receptor kinase	Chemosensation	Fukuto *et al.,* [35]
gpa-2(pk16)	G protein alpha subunit	Dauer formation	Zwaal *et al*., [73]
fat-3(wa22)	Fatty acid desaturase	Polyunsaturated fatty acid metabolism	Watts & Browse, [50]
fat-4(wa14)	Fatty acid desaturase	Polyunsaturated fatty acid metabolism	Watts & Browse, [50]
fat-1(wa9)	Fatty acid desaturase	Polyunsaturated fatty acid metabolism	Watts & Browse, [50]
osm-9(ky10)	Transient receptor potential channel	Mechanosensation and olfactory adaptation	Colbert *et al*., [48]
egl-30(n686)	G protein q alpha subunit	Olfactory adaptation	Matsuki *et al*., [4]
egl-8(n488)	Phospholipase C beta	Synaptic transmission	Miller *et al*., [57]
egl-10(md176)	Regulator of G protein signaling	Olfactory adaptation	Matsuki *et al*., [4]
pkc-1(ok563)	Protein Kinase C	Neuropeptide secretion	Sieburth *et al*., [56]
mbk-1(pk1389)	Mini-brain kinase	Odortaxis	Raich *et al*., [79]
ttx-3(ks5)	LIM homeodomain transcription factor	Regulation of AIY function	Hobert *et al.,* [80]
unc-18(md299)	*S. cerevisiae* SEC1 orthologue	Synaptic vesicle complex protein	Hata *et al*., [81]
che-11(e810)	Intraflagellar transport protein	Ciliogenesis	Perkins *et al*., [29]
che-2(e1033)	WD40 repeat containing protein	Ciliogenesis	Fujiwara *et al.,* [30]
sel-12(ar131)	Presenilin	Development	Kitagawa *et al*., [84]
adp-1(ky20)	Uncloned	Olfactory adaptation	Colbert & Bargmann, [1]
